# Multiomics comparison among populations of three plant sources of Amomi Fructus

**DOI:** 10.1093/hr/uhad128

**Published:** 2023-08-01

**Authors:** Xinlian Chen, Shichao Sun, Xiaoxu Han, Cheng Li, Fengjiao Wang, Bao Nie, Zhuangwei Hou, Song Yang, Jiaojiao Ji, Ge Li, Yanqian Wang, Xiaoyu Han, Jianjun Yue, Cui Li, Wei Li, Lixia Zhang, Depo Yang, Li Wang

**Affiliations:** School of Pharmaceutical Sciences, Sun Yat-Sen University, 510006 Guangzhou, China; Shenzhen Branch, Guangdong Laboratory of Lingnan Modern Agriculture, Key Laboratory of Synthetic Biology, Ministry of Agriculture and Rural Affairs, Agricultural Genomics Institute at Shenzhen, Chinese Academy of Agricultural Sciences, 518120 Shenzhen, China; Shenzhen Branch, Guangdong Laboratory of Lingnan Modern Agriculture, Key Laboratory of Synthetic Biology, Ministry of Agriculture and Rural Affairs, Agricultural Genomics Institute at Shenzhen, Chinese Academy of Agricultural Sciences, 518120 Shenzhen, China; Shenzhen Branch, Guangdong Laboratory of Lingnan Modern Agriculture, Key Laboratory of Synthetic Biology, Ministry of Agriculture and Rural Affairs, Agricultural Genomics Institute at Shenzhen, Chinese Academy of Agricultural Sciences, 518120 Shenzhen, China; Shenzhen Branch, Guangdong Laboratory of Lingnan Modern Agriculture, Key Laboratory of Synthetic Biology, Ministry of Agriculture and Rural Affairs, Agricultural Genomics Institute at Shenzhen, Chinese Academy of Agricultural Sciences, 518120 Shenzhen, China; Shenzhen Branch, Guangdong Laboratory of Lingnan Modern Agriculture, Key Laboratory of Synthetic Biology, Ministry of Agriculture and Rural Affairs, Agricultural Genomics Institute at Shenzhen, Chinese Academy of Agricultural Sciences, 518120 Shenzhen, China; Shenzhen Branch, Guangdong Laboratory of Lingnan Modern Agriculture, Key Laboratory of Synthetic Biology, Ministry of Agriculture and Rural Affairs, Agricultural Genomics Institute at Shenzhen, Chinese Academy of Agricultural Sciences, 518120 Shenzhen, China; Shenzhen Branch, Guangdong Laboratory of Lingnan Modern Agriculture, Key Laboratory of Synthetic Biology, Ministry of Agriculture and Rural Affairs, Agricultural Genomics Institute at Shenzhen, Chinese Academy of Agricultural Sciences, 518120 Shenzhen, China; Shenzhen Branch, Guangdong Laboratory of Lingnan Modern Agriculture, Key Laboratory of Synthetic Biology, Ministry of Agriculture and Rural Affairs, Agricultural Genomics Institute at Shenzhen, Chinese Academy of Agricultural Sciences, 518120 Shenzhen, China; Shenzhen Branch, Guangdong Laboratory of Lingnan Modern Agriculture, Key Laboratory of Synthetic Biology, Ministry of Agriculture and Rural Affairs, Agricultural Genomics Institute at Shenzhen, Chinese Academy of Agricultural Sciences, 518120 Shenzhen, China; Yunnan Key Laboratory of Southern Medicine Utilization, Yunnan Branch Institute of Medicinal Plant Development, Chinese Academy of Medical Sciences, 666100 Jinghong, China; Yunnan Key Laboratory of Southern Medicine Utilization, Yunnan Branch Institute of Medicinal Plant Development, Chinese Academy of Medical Sciences, 666100 Jinghong, China; School of Pharmaceutical Sciences, Sun Yat-Sen University, 510006 Guangzhou, China; Shenzhen Branch, Guangdong Laboratory of Lingnan Modern Agriculture, Key Laboratory of Synthetic Biology, Ministry of Agriculture and Rural Affairs, Agricultural Genomics Institute at Shenzhen, Chinese Academy of Agricultural Sciences, 518120 Shenzhen, China; School of Pharmaceutical Sciences, Sun Yat-Sen University, 510006 Guangzhou, China; School of Traditional Dai-Thai Medicine, West Yunnan University of Applied Sciences, 666100 Jinghong, China; National Center for TCM Inheritance and Innovation, Guangxi Botanical Garden of Medicinal Plants, 530023 Nanning, China; Shenzhen Branch, Guangdong Laboratory of Lingnan Modern Agriculture, Key Laboratory of Synthetic Biology, Ministry of Agriculture and Rural Affairs, Agricultural Genomics Institute at Shenzhen, Chinese Academy of Agricultural Sciences, 518120 Shenzhen, China; Yunnan Key Laboratory of Southern Medicine Utilization, Yunnan Branch Institute of Medicinal Plant Development, Chinese Academy of Medical Sciences, 666100 Jinghong, China; School of Pharmaceutical Sciences, Sun Yat-Sen University, 510006 Guangzhou, China; Shenzhen Branch, Guangdong Laboratory of Lingnan Modern Agriculture, Key Laboratory of Synthetic Biology, Ministry of Agriculture and Rural Affairs, Agricultural Genomics Institute at Shenzhen, Chinese Academy of Agricultural Sciences, 518120 Shenzhen, China; Kunpeng Institute of Modern Agriculture at Foshan, Chinese Academy of Agricultural Sciences, 528200 Foshan, China

## Abstract

Amomi Fructus (Sharen, AF) is a traditional Chinese medicine (TCM) from three source species (or varieties), including *Wurfbainia villosa* var. *villosa* (WVV), *W. villosa* var. *xanthioides* (WVX), or *W. longiligularis* (WL). Among them, WVV has been transplanted from its top-geoherb region, Guangdong, to its current main production area, Yunnan, for >50 years in China. However, the genetic and transcriptomic differentiation among multiple AF source species (or varieties) and between the origin and transplanted populations of WVV is unknown. In our study, the observed overall higher expression of terpenoid biosynthesis genes in WVV than in WVX provided possible evidence for the better pharmacological effect of WVV. We also screened six candidate borneol dehydrogenases (BDHs) that potentially catalyzed borneol into camphor in WVV and functionally verified them. Highly expressed genes at the P2 stage of WVV, *Wv05G1424* and *Wv05G1438*, were capable of catalyzing the formation of camphor from (+)-borneol, (−)-borneol and DL-isoborneol. Moreover, the *BDH* genes may experience independent evolution after acquiring the ancestral copies, and the following tandem duplications might account for the abundant camphor content in WVV. Furthermore, four populations of WVV, WVX, and WL are genetically differentiated, and the gene flow from WVX to WVV in Yunnan contributed to the greater genetic diversity in the introduced population (WVV-JH) than in its top-geoherb region (WVV-YC), which showed the lowest genetic diversity and might undergo genetic degradation. In addition, *terpene synthesis* (*TPS*) and *BDH* genes were selected among populations of multiple AF source species (or varieties) and between the top- and non-top-geoherb regions, which might explain the difference in metabolites between these populations. Our findings provide important guidance for the conservation, genetic improvement, and industrial development of the three source species (or varieties) and for identifying top-geoherbalism with molecular markers, and proper clinical application of AF.

## Introduction

Top-geoherbalism, similar to “*Daodi*” in China and “Provenance” or “Terroir” in Europe, refers to traditional herbs grown in certain native ranges that possess better quality and efficacy than those grown elsewhere [1, 2]. These superior characteristics are attributed to differences in the components and resulting secondary metabolites among plant populations caused by multiple factors, such as genetic elements, environmental factors, and cultural processing [3–5]. The varied efficacy of traditional medicines from multiple sources results in mixtures of inferior and superior products in the market, which inhibits standardization and internationalization of traditional medicine.

One special case of top-geoherbalism is the legally accredited multiple-source plant species for a single medicine. The content of chemical components in different origin species, which are often listed for quality control of traditional Chinese medicine (TCM), are frequently different and thus the pharmacological effect of products with different origins varies [6–9], although they are used as the same TCM clinically. For example, Ephedrae herba is the dry herbaceous stem of *Ephedra sinica*, *E. intermedia, or E. equisetina* [10]. However, the total alkaloid content with the greatest acute toxicity is higher in *E. equisetina* than in the first two species [11, 12]. The main alkaloid accumulated in *E. sinica* with the best effect of relaxation and cough relief was ephedrine, whereas in *E. intermedia*, it was (+)-pseudoephedrine [11, 13, 14]. Another common case of top-geoherbalism is when different populations of the same species demonstrate different pharmacological efficacies. For example, Tan et al. identified the chemical markers β-ocimene, α-pinene, 3-methylbutanal, heptanes, and butanal for distinguishing between Radix *Angelica sinensis* from its top-geoherb regions with superior clinical properties and those from non-top-geoherb regions [15].

**Figure 1:**
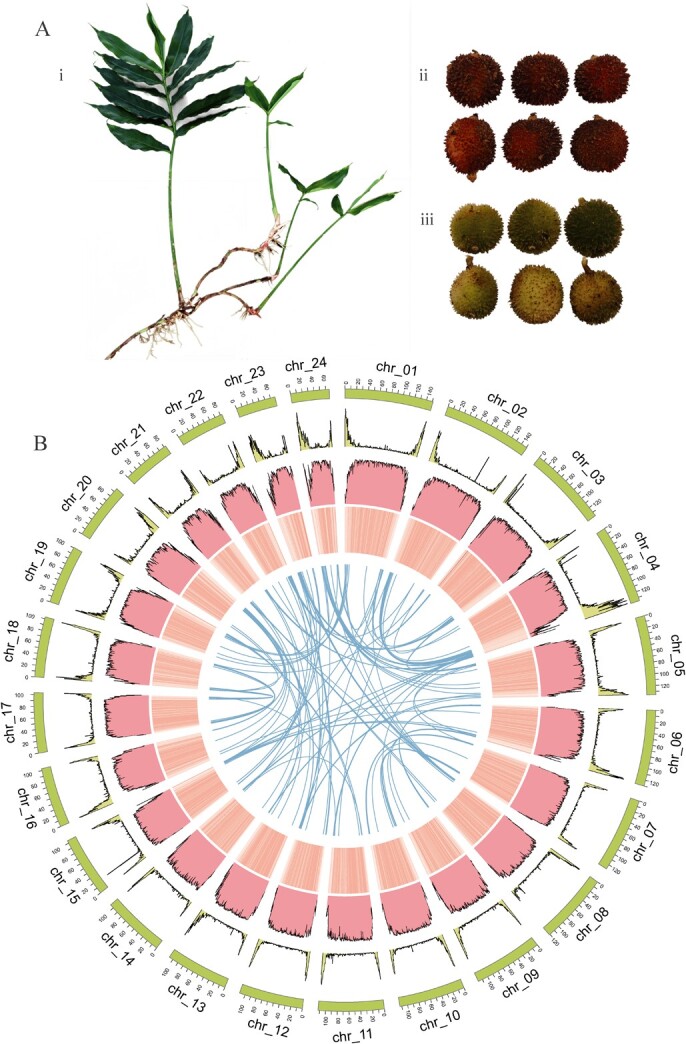
Overview of WVV genome assembly and genomic features. (A) Morphological characteristics of WVV and WVX. (i) Plant and (ii) fruits of WVV and (iii) fruits of WVX. (B) Distribution of WVV genomic features. The circos represented synteny, GC content, TE distribution, gene density and karyotypes from inside to outside, respectively. All these genomic features were calculated with 500 kb non-overlapped sliding windows.

Amomi Fructus (Sharen, AF) provides an ideal system to investigate top-geoherbalism because it is legally recorded from multiple species (or varieties), and the varied populations of the same species possess distinct biochemical components and content. In the Chinese Pharmacopoeia, AF is described as the dry and mature fruit of *Wurfbainia villosa* var. *villosa* (Lour.) Škorničk. & A.D.Poulsen (WVV, Figure 1A), *Wurfbainia villosa* var. *xanthioides* (Wall. ex Kuntze) Škorničk. & A.D.Poulsen (WVX, Figure 1A), or *Wurfbainia longiligularis* (T.L.Wu) Škorničk. & A.D.Poulsen (WL) in the Zingiberaceae family [10]. AF is one of the four most important Southern China medicines and is used as both medicine and food [16–18]; it is well-known for its efficacy of soothing a fetus, stopping diarrhea, and appetizing [10], and the effective components are volatile oils, mainly terpenes, including monoterpenes (e.g. bornyl acetate, borneol, camphor, myrcene, limonene, and α-terpinene) and sesquiterpenes (e.g. germacrene, bicyclogermacrene, α-copaene, and α-santalol) [19].

Profound differences in the content of volatile metabolites of the three plant species (or varieties) of AF have been observed [20–22], which is reflected in the different standards for the content of volatile oils of them in the Chinese Pharmacopoeia. The volatile oil content in the seeds of WVV and WVX is required to be ≥3.0% (ml/g) and in that of WL ≥1.0% (ml/g), respectively [10]. The main volatile components of WVV and WL are bornyl acetate and camphor for WVX [20–22]. From the clustering analysis of volatile metabolites identified by high-performance liquid chromatography (HPLC), WVV and WL showed similar patterns, and they were clearly differentiated from WVX [23]. Marketed AF is sometimes a mixture of WVV and WVX, with a fruit color the same as that of WVV after processing. Thus, the genetic characteristics of the three species (or varieties), including the genomic variation and gene expression underlying the different volatile compounds, await further clarification for the molecular identification of medicinal sources.

In addition, the top-geoherb region of WVV is Yangchun, Guangdong province, China [18]. However, WVV in Yangchun must be artificially pollinated and thus demonstrates poor yields. Since the 1960s, WVV has been gradually introduced to Guangxi, Fujian, and Yunnan provinces, where natural pollination occurs via local insects that greatly increases its yield. Currently, the production of WVV from Yunnan accounts for >80% of the market share, but the price is 20 times lower than that from Yangchun that are labeled “top-geoherbalism” products [18]. However, the efficacy of WVV in Guangdong and Yunnan provinces is controversial. Some researchers have found no significant difference in the pharmacological activities of WVV from the two localities [24], whereas some studies have found that the comprehensive quality score, such as the content of volatile oils and bornyl acetate of WVV in Guangdong was the highest among WVV from other places [25, 26]. Tang et al [27] found that the 2,2-diphenyl-1-picrylhydrazyl scavenging rate and hydroxyl radical scavenging rate of WVV in Guangdong were higher, but the reducing ability of WVV in Yunnan was stronger. Given that the genetic separation of WVV from Guangdong and Yunnan has occurred only over 50 years, the genetic variation of WVV from the top-geoherb and main production areas is obscure.

Genetic studies have been advanced by a recent release of the genome of WVV [28], and many *terpene synthesis* (*TPS*) genes have been screened and verified, revealing parts of the biosynthetic pathway of terpenes in WVV [28–33]. The identified genes included the *linalool synthase* gene (*AvTPS2*), *α-santalene* and *α-bergimonene synthase* gene (*AvTPS15*) [34], and *α-pinene* and *β-pinene synthase* gene (*AvPS*) [35]. The monoterpene bornyl acetate is one of the most significant characteristic substances in WVV. A previous study revealed that *WvBAT3* and *WvBAT4* might be the two key borneol acetyltransferases in the *BAHD* gene family because they are synthesized in the seeds of WVV [28]. However, the enzymes catalyzing borneol to camphor have not been resolved in WVV. According to the chemical structures of borneol and camphor, this oxidation step is possibly catalyzed by borneol dehydrogenase (BDH). *BDH* genes have been cloned and functionally verified in several species, such as *CcBDH3* in *Cinnamomum camphora* [36], *LiBDH* in *Lavandula x intermedia* [37], and *AaBDH* in *Artemisia annua* [38]. They belong to the short-chain dehydrogenases/reductases subfamily [37, 39], constituting a large family of NAD(P)(H)-dependent oxidoreductases that share sequence motifs and have similar mechanisms [40, 41]. Taken together, BDH catalyzing borneol to camphor in WVV await further exploration.

This study aimed to *de novo* assemble a chromosome-level genome of WVV from its top-geoherb location and conducted a comprehensive comparison among populations of the three plant species (or varieties) genetically and transcriptionally, and between the top-geoherb and main production areas of WVV. We planned to investigate the following: 1) the expressional difference of terpenes-related genes between WVV and WVX; 2) the BDHs catalyzing borneol into camphor in WVV; and 3) the genetic differentiation among populations of WVV, WVX, and WL. The overall goal was to support important guidance for the conservation and genetic improvement of AF source species (or varieties) and for identifying top-geoherbalism and proper clinical applications of AF.

## Results

### Genome sequencing, assembly, and annotation of *W. villosa* var. *villosa*

The genome size of WVV was estimated to be ~2.62 Gb by flow cytometry, which gave us a rough idea of the amount of sequencing data required to produce a good quality *de novo* assembled genome. In total, we obtained 157.07 Gb (~55.50X) of high-fidelity (HiFi) long reads and 286.28 Gb (~101.16X) of high-throughput chromosome conformation capture (Hi-C) short reads. We obtained a haplotype-resolved genome assembly at the contig level. Haplotype 1 had 2901 contigs (N50 = 8.30 Mb) with a total size of 2.83 Gb (Table S1). The genome size of haplotype 2 was 2.77 Gb with a contig N50 of 7.01 Mb (Table S1). Subsequently, the haplotype 1 with a larger genome size and higher contig N50 was chosen for the scaffold assembly and following analysis; its contig sets were anchored to 24 pseudochromosomes on the basis of Hi-C contacts with a scaffolding rate of 94.2% (Figure S1). The final assembled WVV genome was 2.83 Gb, and the scaffold N50 was 112.8 Mb (Table 1). The length of the 24 pseudochromosomes ranged from 151 371 763 (chr_01) to 66 131 911 bp (chr_24) (Figure 1B and Table S2). The genome size of WVV was almost the same as the published WVV genome size (2.80 Gb) [17] and 1.4–2.9 times larger than that of other Zingiberaceae species [42–45]. The average GC content of the WVV genome was 40.73% (Table 1). To test the quality of the WVV genome assembly, RNA-seq paired-end reads were mapped to the assembled genome with mapping rates of 92.08%–96.61%. In addition, Benchmarking Universal Single-Copy Orthologs assessment (BUSCO) analysis showed that the assembled genome covered 99.8% of the viridiplantae orthologous gene set (Table 1).

**Table 1:** Comparison of the two WVV genomes

	This study	Yang et al [17]
**Contig**		
Total assembly size (Mb)	2834.2	2799.2
Total contig number	2901	1100
Maximum contig length (bp)	56 657 728	-
Contig N50 length (Mb)	8.3	9.1
Contig N90 length (bp)	1 378 773	-
**Scaffold**		
Total size (Mb)	2669.5	2575.5
Maximum scaffold length (bp)	151 371 763	139 364 153
Scaffold N50 (Mb)	112.8	109.9
Scaffold N90 (bp)	68 159 171	-
GC content (%)	40.7	40.2
Complete BUSCOs (%)	99.8	97.9
Mapping rate of Illumina resequencing data (%)	99.42	98.25
**Annotation**		
Number of genes	42 473	42 588
Average coding-sequence length (bp)	965	1192
Repeat content (%)	85.67	87.23
LTR (%)	79.68	78.26
GO scale (%)	40.5	44.9
KEGG scale (%)	40.5	28.5
Number of *TPS* genes	90	66

Combining the *ab initio* prediction and orthologous protein and transcriptomic data, we annotated 42 473 coding genes, of which 41 506 genes were located on 24 pseudochromosomes. The previous genome annotated 42 588 genes and 30 188 were on pseudochromosomes [17]. The average gene density was one gene per 66.73 kb, with the genes unevenly distributed, being more abundant toward the chromosomal ends and similar to the published one [17] (Figure 1B). The average length of coding sequences of the predicted genes was 965 bp, with an average of 4.22 exons per gene (Table S3). Approximately 78.73% of the protein-coding genes were functionally annotated by searching the SwissProt (69.61%), Kyoto Encyclopedia of Genes and Genomes (KEGG) (40.5%), Pfam (74.0%), and Gene Ontology (GO) databases (40.5%). Transposable element (TE) sequences comprised 85.67% (2.43 G) of the WVV genome. Among them, long-terminal repeat (LTR) was the most abundant repetitive type (79.68%), and *Copia* was the largest group in the LTR, accounting for 50% of the entire genome (Table S4). We found a higher mapping rate of Illumina resequencing data to our PacBio HiFi assembly than that of the published ONT assembly (99.42% vs. 98.25%; Table 1), which indicated a more accurate assembly of our WVV genome. On the other hand, despite the comparable scaffold/contig N50 size of the two assemblies, our assembled genome has largely increased the gene anchoring rate (~97% compared to ~70% predicted genes anchored onto chromosomes in the previous one), suggesting a higher completeness of assembly.

### Comparison of the expression levels of terpenoid biosynthetic genes between *W. villosa* var. *villosa* and *W. villosa* var. *xanthioides*

**Figure 2:**
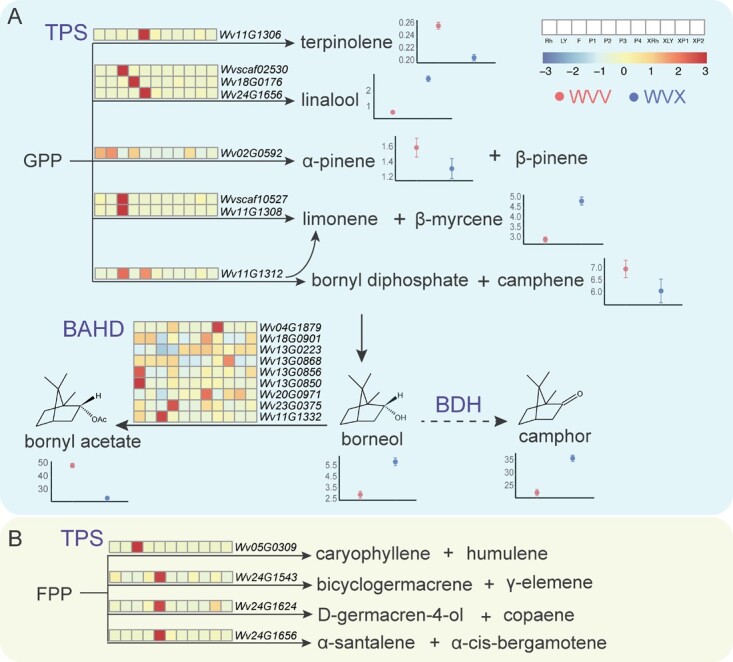
The heatmap of terpene synthesis-related gene expressions from different tissues and fruit developmental stages in WVV and WVX. The heatmap represented rhizomes (Rh), young leaves (LY), flowers (F), fruits of 10-day after flowering (P1), 30-day after flowering (P2), 60-day after flowering (P3), 90-day after flowering (P4) of WVV, and rhizomes (XRh), young leaves (XLY), 75-day after flowering (XP1), 90-day after flowering (XP2) of WVX from left to right. On the right or below of the chemical structure was the difference in the relative content of each volatile oil in the fruits reported in Ao et al. [21] The x-axis showed WVV (red) and WVX (blue). The y-axis represented the relative content. (A) Monoterpene synthesis. (B) Sesquiterpene synthesis.

The content of certain secondary metabolites between WVV and WVX was obviously different according to previous studies (Figure 2A) [21]. The content of terpinolene, α-pinene, camphene, and bornyl acetate was higher in WVV, whereas linalool, β-myrcene, borneol, and camphor were richer in WVX. Terpenoid biosynthesis in WVV and WVX mainly included three types of genes: *TPS*, *BAHD*, and *BDH*. To investigate the pattern of gene expression affecting the content of volatile compounds between the two varieties, we analyzed the expression of genes involved in multiple terpene biosynthesis in various tissues and fruit developmental stages between WVV and WVX (see Materials and Methods; Figure 2).

Subsequently, we constructed a phylogenetic tree using *TPS* genes from the WVV genome and the functionally verified *TPS* genes in a previous study [17]. Based on the tree, 11 *TPS* genes that clustered with the verified ones were selected (Figure S2). The transcriptome data were generated for the rhizomes (Rh), young leaves (LY), flowers (F), fruits of 10-day after flowering (P1), 30-day after flowering (P2), 60-day after flowering (P3), and 90-day after flowering (P4) of WVV and rhizomes (XRh), young leaves (XLY), fruits of 75-day after flowering (XP1), and 90-day after flowering (XP2) of WVX to compare the differences in expression between the two varieties. Overall, the expression levels of these *TPS* genes were relatively higher in WVV than in WVX (Figure 2), and high expression of *TPS* genes did not always coincide with the metabolite content trends in WVV and WVX. For the monoterpene biosynthesis genes, the highest expression level of *Wv11G1306* was observed at the P2 stage, consistent with the higher content of terpinolene in the fruits of WVV (Figure 2A). High expression of the *TPS* genes *Wv18G0176* and *Wv24G1656* (for linalool) appeared at the P1 and P2 stages, respectively, in contrast to the trend of the content of linalool in WVV and WVX. *Wv02G0592*, *Wvscaf10527*, *Wv11G1308*, and *Wv11G1312* were all bi- or multiproduct enzyme genes as determined on the basis of verified homologous genes [17]. We only knew the content trends of α-pinene, β-myrcene, and camphene of WVV and WVX, not those of β-pinene, limonene, and bornyl diphosphate [21]. The gene expression levels of *Wv02G0592* (for α-pinene and β-pinene), *Wv11G1312* (for limonene, bornyl diphosphate, camphene), and *Wvscaf10527* and *Wv11G1308* (for limonene and β-myrcene) showed low expression in the fruits of both varieties. In addition, for sesquiterpenoids, Wv24G1543 catalyzing the substrate to become bicyclogermacrene and γ-elemene, Wv24G1624 to become D-germacren-4-ol and copaene, and Wv24G1656 to become α-santalene and α-cis-bergamotene were all expressed at higher levels in the P2 stage of WVV (Figure 2B). The inconsistency between the expression levels of *TPS* genes and its encoding enzymes corresponding catalytic products content could be related to multiple factors: 1) the lack of collection of younger fruit in WVX, missing the high-expression period; 2) the different sources of expression and secondary metabolite data; 3) the potential transportation of secondary metabolites from their biosynthetic tissues to other tissues [46, 47]; 4) the content of metabolite is related to the expression level of its bona fide enzyme, but not with other copies of its sequence homologs [28]; 5) some *TPS* genes are multifunctional (i.e. able to produce multiple secondary metabolites [17, 36]), which makes the correlation of gene expression levels and one of its products insignificant.

Borneol, bornyl acetate, and camphor are the three most significant chemical substances in WVV and WVX, with borneol as the same substrate, bornyl acetate was produced by BAHD and the camphor by BDH. We located nine *BAHD* genes in this genome according to experimentally verified *BAHD*s [28] (Figure 2A). The higher expression levels of *Wv18G0901*, *Wv13G0223*, and *Wv20G0971* at the P4 stage and of *Wv23G0375* at the P1 stage may be the key candidate genes (Figure 2A).

To further explore the regulation of biosynthetic genes, 2781 transcription factors (TFs) were identified in the WVV genome. To identify the TFs regulating bornyl acetate biosynthesis in WVV, we constructed a gene co-expression network. We further analyzed TFs regulating the above-mentioned *BAHD* genes and found that 189, 278, 81, 203, 92, 1, and 276 TFs were involved in regulating *Wv04G1879*, *Wv11G1332*, *Wv13G0223*, *Wv13G0850*, *Wv13G0856*, *Wv13G0868*, and *Wv23G0375* genes, respectively (Figure S3 and Table S7). The well-known *MYB* and *WRKY* TFs were involved in regulating bornyl acetate biosynthesis (Figure S3). Previous studies showed that *MYB* and *WRKY* TFs participated in regulating the synthesis of terpenes [48–51], and in particular, *WRKY *was found to have an important role in bornyl acetate biosynthesis [32]. This analysis provided a list of TF candidates for further functional verification in bornyl acetate biosynthesis.

### Candidate BDHs catalyzing borneol into camphor and enzyme activity assay *in vivo*

**Figure 3:**
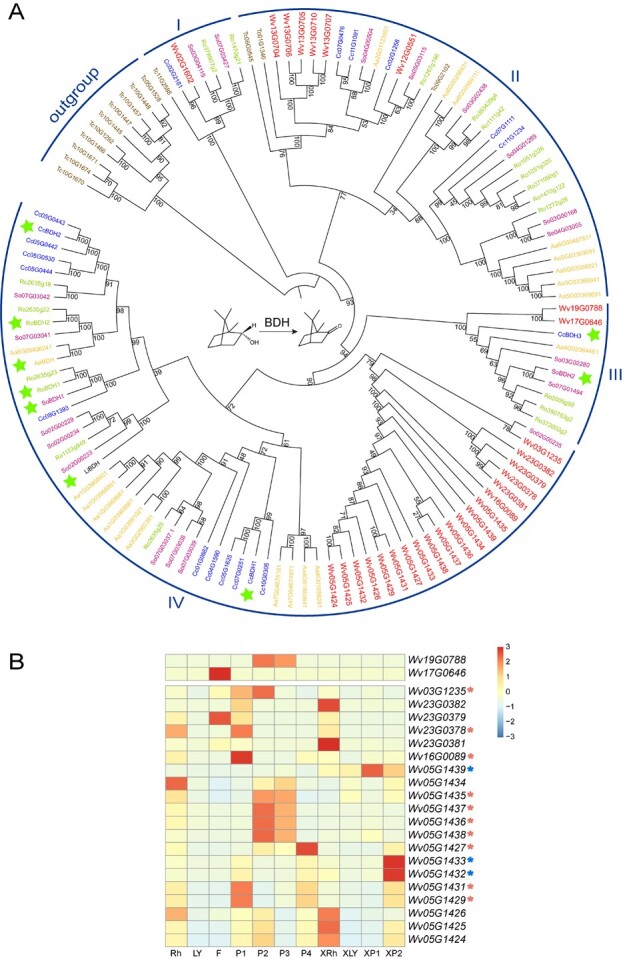
Phylogenetic tree of BDH homologous proteins in various species. (A) The phylogenetic tree of BDH genes. Wv: WVV*.* Cc: *Cinnamomum camphora*. So: *Salvia officinalis*. Tc: *Taxus chinensis*. Aa: *Artemisia annua*. Ro: *Rosmarinus officinalis*. The green stars marked the verified BDH enzymes. The numbers on the branches showed the bootstraps. (B) The heatmap of expression level of candidate *BDH* genes identified in clades III and IV in different tissues and developmental periods of WVV and WVX. Red asterisks indicated ten *BDH* genes, which were highly expressed in WVV fruit developmental stages, and blue asterisks showed three *BDH* genes, which were highly expressed in WVX fruit developmental stages.

To investigate the evolutionary relationships among *BDH* genes and identify candidate genes in WVV, we used the maximum likelihood method to construct a phylogenetic tree. The tree contained BDH proteins from one monocot (WVV), one gymnosperm (*Taxus chinensis* [52], as the outgroup), one magnoliid (*C. camphora* [2]), and three eudicots (*Salvia officinalis* [53], *A. annua* [54], and *Rosmarinus officinalis* [55]), and the numbers of BDHs in the above-mentioned species were 29, 14, 16, 19, 20, and 18, respectively. Nine experimentally verified BDH proteins that able to catalyze borneol into camphor, from five species were also included (Figure 3A; Supplementary Data 1; Table S5). In total, the tree included 125 BDHs from seven species, among which only one was from *L. intermedia*, LiBDH, owing to the absence of its genome.

Except for the 10 BDH proteins of the outgroup, we divided the remaining members into four clades from I to IV, and the bootstrap of each clade was >75 (Figure 3A). The BDH protein numbers from clades I to IV were 7, 36, 10, and 62, respectively. Only clades I and II had BDH proteins from gymnosperm, suggesting that clades III and IV may have originated from these two clades and BDH proteins in clades I and II appeared before the divergence between gymnosperm and angiosperm. The BDH proteins of each species were dispersed in different clades with clades I to IV containing 5, 6, 5, and 6 species, respectively, suggesting that these species underwent independent evolution after acquiring ancestral copies. Clades I to IV included 1, 6, 2, and 20 BDHs of WVV, respectively (Figure 3A). Clade III comprised Wv19G0788 and Wv17G0646 and two validated enzymes, CcBDH3 and SoBDH2. Clade IV consisted of 20 BDHs in WVV and seven experimentally-validated BDH proteins: CcBDH1, LiBDH, SoBDH1, RoBDH1, AaBDH, RoBDH2, and CcBDH2. Thus, we speculated that the 22 BDH proteins in clades III and IV were more likely candidate enzymes that catalyzed the dehydrogenation of borneol to form camphor in WVV. Interestingly, we found that the WVV *BDH* genes of clade IV were compactly distributed on chromosomes 23 and 5, suggesting a couple of tandem duplication events, which were also reflected in the BDHs from *T. chinensis*, *A. annua*, and *C. camphora* (Figure 3A).

To further narrow down the candidate *BDH* genes in WVV, we examined the expression levels of the 22 candidate *BDHs* in various tissues and fruit developmental stages (Figure 3 and Table S6). For clade III, *Wv19G0788* was highly expressed at both P2 and P3, and *Wv17G0646* was lowly expressed at any fruit stage. However, the expression levels of *Wv19G0788* and *Wv17G0646* in all tissues were almost zero, so they were not likely candidate genes (Table S6). For clade IV (20 WVV *BDH* genes), 10 genes (*Wv03G1235*, *Wv23G0378*, *Wv16G0089*, *Wv05G1435*, *Wv05G1436*, *Wv05G1437*, *Wv05G1438*, *Wv05G1427*, *Wv05G1431*, and *Wv05G1429*) were at least highly expressed in one fruit stage, making them more likely candidate *BDH* genes in the fruits of WVV (Table S6). In addition, *Wv05G1439*, *Wv05G1433*, and *Wv05G1432* were only highly expressed in the fruit stages of WVX, but not in WVV. The expressional differences between the two groups of genes may be the underlying reason for the difference in camphor content between WVV and WVX.

**Figure 4:**
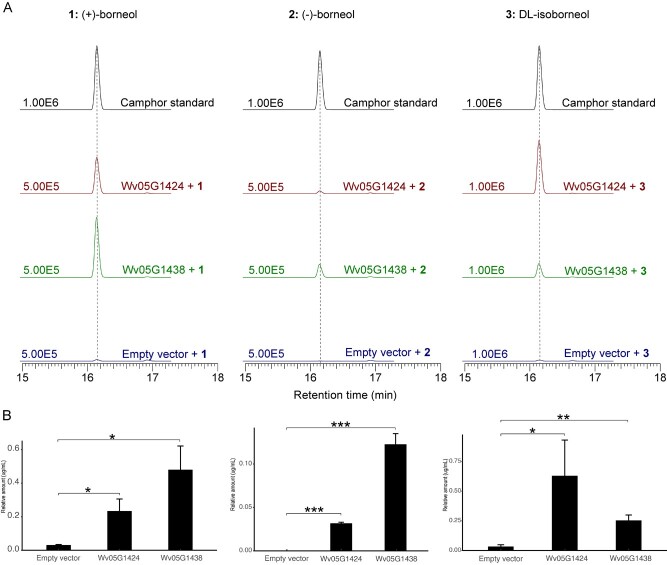
Enzyme activity assay *in vivo* of Wv05G1424 and Wv05G1438. (A) The gas chromatography–mass spectrometry (GC–MS) chromatogram of the *in vivo* reaction products. The substrate of Wv05G1424 and Wv05G1438 was (+)-borneol (**1**), (−)-borneol (**2**), and DL-isoborneol (**3**), respectively. (B) The relative amount (μg/mL) of camphor yielded by empty vector, Wv05G1424 and Wv05G1438 enzyme at the same substrate concentration (100 μM) of (+)-borneol, (−)-borneol, and DL-isoborneol from left to right, respectively. Error bars represent the standard deviation (n = 3) (*P < 0.05, **P < 0.01, ***P < 0.001; paired *t*-test).

Based on the phylogenetic tree using BDHs and previously reported BDHs [36] that can catalyze the conversion of (+)-borneol, (−)-borneol, or DL-isoborneol in plants, the gene expression correlation between *BDHs* and *BPPS* (*Wv11G1312*, the verified upstream camphor synthetic gene) [33], as well as the expression level of *BDH* genes, a total of six BDHs (Wv05G1424, Wv05G1435, Wv05G1438, Wv13G0706, Wv16G0089, Wv03G1235) were selected for *in vivo* enzyme activity analysis in tobacco. The results indicated that two BDHs (Wv05G1424 and Wv05G1438), which were highly expressed at the P2 stage of WVV, could catalyze the formation of camphor from (+)-borneol, (−)-borneol and DL-isoborneol (Figure 4). However, the other four BDHs were unable to catalyze any of the three substrates. Unexpectedly, we found that camphor could also be detected in tobacco when we injected empty vector and fed (+)-borneol or DL-isoborneol without BDH enzymes, indicating that tobacco may contain BDH enzymes that can catalyze the production of camphor from (+)-borneol and DL-isoborneol or it is possible that the process could happen spontaneously. When the substrate was (−)-borneol, we did not detect any camphor in the empty vector. However, the camphor content significantly increased 4–19 times (paired *t*-test，*p* < 0.05) for the substrates of (+)-borneol and DL-isoborneol when added with Wv05G1424 or Wv05G1438 enzymes compared to that with empty vector. The results suggested that Wv05G1424 and Wv05G1438 can efficiently catalyze the formation of camphor from (+)-borneol, (−)-borneol, and DL-isoborneol (Figure 4).

We further explored the expression regulation of the *BDH* genes *Wv05G1424* and *Wv05G1438*, and they were regulated by 17 and 112 TFs, respectively (Figure S4 and Table S8), including the *bHLH*, *ERF*, *WRKY*, *NAC*, and *MYB* families, which have been reported to be crucial in plant growth and development, stress resistance, and secondary metabolism [56, 57]. In addition, *Wv05G1424* genes was regulated by *RAV* while *Wv05G1438* was not. *Wv05G1438 wa*s also regulated by more unique TFs (not regulating *Wv05G1424*), such as *AP2*, *bZIP*, *C2H2*, *Dof*, *WOX*. Further experiments are needed to verify the regulation role of those TFs.

### Resequencing of Amomi Fructus populations

To explore genetic variation of the three source species (or varieties) of AF, leaf tissues from 39 accessions, which contained 20 samples of WVV (10 WVV-YC from Yangchun, Guangdong province, and 10 WVV-JH from Jinghong, Yunnan province), 10 WVX and nine WL were used for the construction of 150-bp paired-end libraries and then sequenced separately. In total, 1.94 Tb (10.0X–29.5X) of raw data were obtained, and the average sequencing depth was 17.5X. The Illumina mapping rate of each population was 98.87%–99.42%, and the numbers of fixed mutations of WVV-JH, WVX, and WL were 25 282, 2 052 511, and 11 754 365, respectively (Table S9). WL demonstrated the lowest mapping rate and the highest number of fixed mutations compared to the reference genome (Table S9), which supports the previous treatment that WL is an independent species based on molecular phylogeny and morphological characteristics [58].

**Figure 5:**
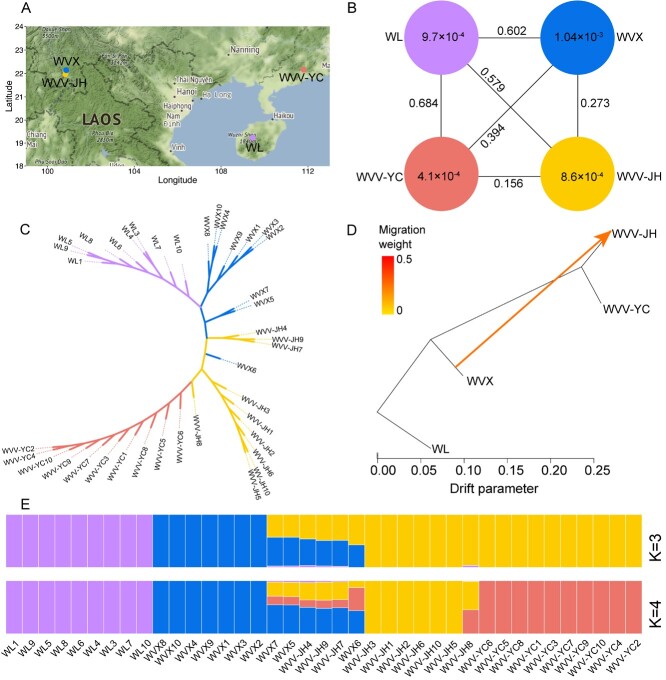
Population genetic analysis of three source species (or varieties) of Amomi Fructus. The color code for the populations is consistent in the figure: purple (WL), red (WVV-YC), blue (WVX) and orange (WVV-JH). (A) Geographical distribution of four populations, including WVV-YC, WVV-JH, WVX, and WL. (B) Population differentiation *F*_ST_ among populations and the nucleotide diversity π of each population based on 24 854 114 SNPs. (C) A NJ tree of 39 accessions based on 160 967 high-quality SNPs. (D) The gene flow from WVX to WVV-JH identified in the TreeMix analyses. The arrow indicates the migration direction. (E) Population structure analyses showed the differentiation of 39 accessions.

The resequenced individuals were collected from three geographic locations (Figure 5A). After mapping to the assembled WVV genome, we identified 24 854 114 putative single-nucleotide polymorphisms (SNPs). We characterized the genetic relationships among WVV-YC, WVV-JH, WVX, and WL with a neighbor-joining (NJ) phylogenetic tree (Figure 5C) and principal component analysis (PCA) (Figure S5). The three origin species (or varieties) were mainly divided into four genetic groups corresponding to the four sampled populations. Based on the filtered SNP dataset (see “Methods”), the optimal number of populations in the STRUCTURE analysis was *K* = 3 (Figure S6). At *K* = 3, one cluster had all WL samples, the second had seven WVX, and the third had 17 WVV (ten from YC, seven from JH), with three individuals of WVV-JH and three of WVX exhibiting an admixture (the percentage of the minor component was >50%; Figure 5E). At *K* = 4, WVV was further divided into a WVV-YC group and a WVV-JH group, the former comprising ten individuals from Yangchun (the top-geoherb location of WVV) and the latter comprising seven individuals from Jinghong, which were introduced from Yangchun approximately 50 years ago.

To study the genetic differentiation among and within the three species (or varieties) of AF, we estimated the population differentiation, *F*_ST_, between populations and the nucleotide diversity, π, within species. The π of the WVX population was estimated on the basis of the high-density SNP data to be 1.04 × 10^−3^, slightly higher than that of the WL (π = 9.7 × 10^−4^) and WVV-JH (π = 8.6 × 10^−4^) populations, and genetic diversity was higher for these three populations than for the WVV-YC (π = 4.1 × 10^−4^) (Figure 5B), suggesting a genetic bottleneck of the narrowly distributed top-geoherbalism population. It was worth noting that the nucleotide diversity in WVV-JH (π = 8.6 × 10^−4^) was more than twice that in the origin location of WVV-YC (π = 4.1 × 10^−4^), which could result from the gene flow with the locally occurring WVX (Figure 5D). To test this hypothesis, we removed the three admixed individuals of WVV-JH; the diversity of WVV-JH was reduced to 5.0 × 10^−4^ but was still higher than that in WVV-YC. This result suggested that human-mediated pollination could not fully compensate for inbreeding in WVV-YC owing to the small population size; further, the introduced population in Yunnan, independent of human-mediated pollination, might have a higher rate of outcrossing and thus restore the genetic diversity.

The highest *F*_ST_ (0.684) was between WL and WVV-YC, followed by that between WL and WVX (*F*_ST_ = 0.602) and that between WL and WVV-JH (*F*_ST_ = 0.579) given that WL and WVV/WVX were obviously different species. The middle rank was between WVX and WVV-YC (*F*_ST_ = 0.394) and between WVX and WVV-JH (*F*_ST_ = 0.273). WVV-JH and WVX were from similar geographical environments, resulting in a higher *F*_ST_ between WVX and WVV-YC. Finally, the lowest *F*_ST_ (0.156) was between WVV-YC and WVV-JH, which was still higher than expected given the short introduction history of WVV-JH. In total, we found that WL was distantly related to both WVV and WVX, and WVX was much more closely related to WVV-JH than to WVX-YC (Figure 5B).

### The *TPS* and *BDH* genes were selected based on population resequencing

To reveal the genetic basis of the difference in the medicinal components of the three AF source species (or varieties), we identified 1065 candidate genes in selective sweep regions between WVV and WVX and 5381 candidate genes between WVV to WL, of which 646 were overlapping (Figure S7 and Tables S10–S13). Usually, the pharmacological effect of AF source species (or varieties) WVV, WVX, and WL decreases successively, and terpenes are the main medicinal components of AF [59, 60]. GO analysis of 646 selected overlapping candidate genes revealed enrichment of genes involved in terpenoid biosynthesis, such as isoprenoid biosynthetic process, isoprenoid metabolic process, terpene metabolic process, and terpene synthase activity (Figure S8), indicating that the selected terpenoid genes were most likely medicinal-related key genes.

**Figure 6:**
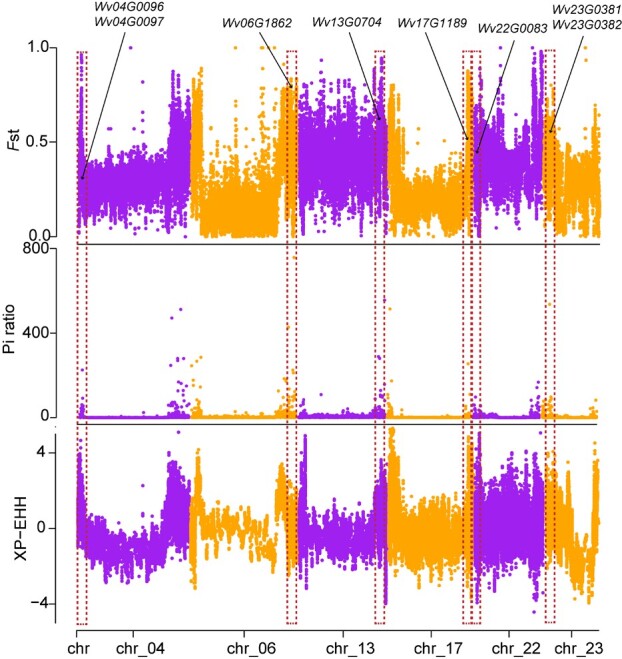
The selective sweep regions identified by at least two statistics among *F*st, π ratio, and XP-EHH methods. Here was comparison of WVV-JH *vs.* WVV-YC. Red dotted boxes and black arrows showed the position of selected genes.

In common recognition, the top-geoherbalism of TCM has a great effect on the quality of Chinese medicinal materials. We identified 3937 selected genes in the comparison of WVV-JH (non-top-geoherb region) with WVV-YC (top-geoherb region) (Figure S9), and based on gene annotation and studies of homologous genes in *Arabidopsis thaliana* or tomato, we inferred selection of genes involving terpenoid biosynthesis (e.g. *TPS*: *Wv04G0096*, *Wv04G0097*, *Wv06G1862*, *Wv17G1189*, and *Wv22G0083*) and camphor biosynthesis (e.g. *BDH*: *Wv13G0704*, *Wv23G0381*, and *Wv23G0382*) (Figure 6). Whether or not these selected genes affect gene expression and subsequent camphor content in the two populations must be determined in further experimental studies.

Compared with WVV, WVX has a very low yield and inferior effects and is often mixed with WVV for sale [61]. To identify molecular markers for the differentiation of the two varieties, we detected 18 268 nonsynonymous SNPs (involved in 9565 genes) in WVV and 707 nonsynonymous SNPs (representing 453 genes) in WVX (Table S14 and Table S15). These SNPs provided valuable molecular markers to distinguish WVX from WVV.

## Discussion

The high-quality assembly of the WVV genome further enriched our understanding of the fundamental biology of this species and promoted future comparative-genomic, genetic-mapping, and gene-cloning studies. Our study revealed that the overall expression of related terpenoid biosynthesis genes in WVV was higher than that in WVX, which provided evidence for the better pharmacological effect of WVV. We screened candidate BDH enzyme that potentially catalyzed borneol into camphor in WVV, and Wv05G1424 and Wv05G1438 were found to catalyze the formation of camphor in tobacco. *BDH* genes may experience independent evolution after acquiring the ancestral *BDH* genes followed by subsequent tandem duplications in WVV. Furthermore, from the perspective of whole genome resequencing data, four populations, including WVV-YC, WVV-JH, WVX, and WL are genetically differentiated. The gene flow from WVX to WVV-JH contributed to the higher genetic diversity in the introduced population of WVV in Yunnan (WVV-JH) than in its top-geoherb region (WVV-YC), which might undergo genetic degradation. Taken together, our study provides new insights into the metabolite biosynthesis, conservation, and industrial development of this medicinal material.

Natural borneol, exhibiting better effects than synthetic borneol owing to the higher proportion of (+)-borneol, is a common valuable composition that is widely applied in TCM formulas and daily chemical products for restoring consciousness, removing heat, and relieving pain [10, 62–65]. How to quickly and obtain great quantities of natural borneol is a matter of great industrial value. *BAHD* and *BDH* genes take borneol as a substrate to produce bornyl acetate and camphor, respectively. Liang et al. revealed the biosynthetic pathways of bornyl acetate in WVV [28]. However, *BDH*s in WVV have not been studied. We found the tandem duplications of *BDH*s in WVV, *T. chinensis*, *A. annua*, and *C. camphora*, which possibly increase its expressional dosage and thus elevate the abundance of metabolites [66, 67]. We observed 4 and 14 tandemly duplicated *BDH* genes in WVV on chromosomes 23 and 5, respectively, possibly contributing to the production of camphor. The expressional difference of the selected ten BDHs, catalyzing borneol into camphor, and Wv05G1439, Wv05G1433, Wv05G1432 might account for the difference in camphor abundance in the fruits of WVV and WVX. More important, we confirmed that Wv05G1424 and Wv05G1438 can catalyze the varied chirality of borneol into camphor *in vivo*. This provides foundation for camphor production and enzyme engineering *in planta*.

WVV originated in Guangdong and was then transplanted into Yunnan in the 1960s [18]. Interestingly, with such a short cultivation time (~50 years), the Yunnan population was genetically differentiated and possessed two-times higher genetic diversity than that of the top-geoherbalism population, which exhibited the lowest genetic diversity. Tang et al. [68] also identified differences between metabolic profiles in Guangdong and Yunnan through ultra-performance liquid chromatography–quadrupole time-of-flight mass spectrometry non-targeted metabolomics and obtained eight metabolites that were expected to be able to differentiate them. The gene flow from the locally adapted variety WVX, which showed the highest genetic diversity among the three species (or varieties), contributed to the increased genetic diversity.

However, one limitation of our study was the absence of a comprehensive evaluation of the volatile components in WVV-YC and WVV-JH when planted in the same environment, which limited our extrapolation to determine if the selected *BDH* genes during the introduction were related to the camphor content and our inference of the potential pharmacological effects of the two populations.

Our results raised concerns about the declining genetic diversity of the top-geoherbalism population with a narrow geographic distribution of some medicinal plants. Decreased genetic diversity implies that species will gradually accumulate deleterious mutations and are more susceptible to serious population shrinkage due to the effect of diseases and insect pests and thus lose the top-geoherbalism advantage [69, 70]. At the same time, our study also pointed out that one possible conservation route is to transplant the plants from the top-geoherb region to other suitable habitats with its close wild relatives co-occurring, as hybridization with the wild relatives with higher genetic diversity will remedy the decreased genetic diversity of the species and provide rich genetic resources for the breeding of medicinal plants. Our study highlights the need to collect and preserve medicinal plant germplasm resources to enhance the environmental adaptability of TCM [71, 72].

## Materials and methods

### Genome sequencing, assembly, and annotation

*Wurfbainia villosa* plants were collected from its top-geoherb regions in Yangchun (111°46′48′′ E, 22°9′36′′ N) in Guangdong province, China. A DNeasy Plant Mini Kit (Qiagen, Germany) was used to extract genomic DNA from fresh young leaves. A 50-μg amount of high-quality DNA was taken to construct SMRTbell™ libraries and sequenced in circular consensus sequencing (CCS) mode on the PacBio Sequell II platform. Hi-C libraries were constructed from the fresh leaves of WVV and then sequenced on the Illumina NovaSeq 6000 platform [73].

The genome assembly of WVV was analyzed by integrating CCS and Hi-C reads via Hifiasm v0.15.1-r334 with the default parameter [74]. Juicer software [75] was used to map the Hi-C sequenced reads to the contig-level assembly of WVV, and then 3D-DNA pipeline [74] was used to correct misjoins, orientation, and order and then generate a draft chromosome assembly. Finally, the draft assembly was visualized in Juicebox Assembly Tools (https://github.com/aidenlab/Juicebox), and manual correction was performed to obtain the chromosome-level genome of WVV. BUSCO v5.1.2 was used to evaluate the completeness of the genome [76].

The EDTA pipeline [77] was used to identify TE in the WVV genome, and the MAKER2 pipeline [78] was applied to predict the coding gene structure from *ab initio* predictions, homolog proteins, and transcriptome data. Functional annotations of coding sequences were aligned by BLASTP (“-e-value 1e–5”) in SwissProt databases and annotated by use of the online EGGNOG-MAPPER (http://eggnog-mapper.embl.de/) for Pfam, GO, and KEGG.

### RNA sequencing

Plant materials of WVV, including rhizomes (Rh), young leaves (LY), flowers (F), fruits of 10-day after flowering (P1), 30-day after flowering (P2), 60-day after flowering (P3), 90-day after flowering (P4), and WVX, including rhizomes (XRh), young leaves (XLY), fruits of 75-day after flowering (XP1), 90-day after flowering (XP2) were used for RNA sequencing, and three biological replicates for each sample.

A RNAprep Pure Plant kit (TIANGEN, China) was used to extract total RNA, and 20 μg RNA was taken for reverse transcription to synthesize cDNA. RNA sequencing was performed on Illumina NovaSeq 6000 platform. HISAT2 software was used to map all clean reads to the WVV genome, and the counts from featureCounts were used to calculate the transcripts per million reads to measure the expression level [79, 80].

### Transcriptional regulation of bornyl acetate and camphor biosynthesis

To identify transcriptional regulatory networks between bornyl acetate, the camphor biosynthetic gene, and TFs, a series of gene expression and co-expression network analyses were performed. Differentially expressed genes in different tissues and weighted correlation network analysis were used to construct a co-expression network [81]. The co-expression network modules were attained and PlantTFDB was used with default parameters to identify TFs in the WVV genome [82]. The networks between genes and TFs were visualized in Cytoscape [83].

### Phylogenetic analysis of *BDH* genes that catalyzed borneol into camphor

To identify candidate *BDH* genes that could convert borneol into camphor, a total of 116 homologous proteins were identified through querying in WVV, *C. camphora*, *S. officinalis*, *T. chinensis*, *A. annua*, and *R. officinalis*. Then, combined with nine experimentally-validated BDH enzymes previously, the phylogenetic tree was constructed, and iTOL was used to visualize and edit the tree [84].

### *In vivo* enzyme activity assay of candidate *BDH* genes

Six candidate *BDH* genes (*Wv05G1424*, *Wv05G1435*, *Wv05G1438*, *Wv13G0706*, *Wv16G0089*, and *Wv03G1235*) were amplified from cDNA of the mixture of leaves and fruits of WVV, and ligated into the pEAQ-HT plasmid. The recombinant plasmids were constructed using the ClonExpress Ultra One Step Cloning Kit (Vazyme, Nanjing, China), followed by transformation into competent JM109. After verification by sequencing, recombinant plasmids were transferred into *Agrobacterium tumefaciens* GV3101 by the freeze–thaw method, and the bacteria were grown on LB plates (50 mg/mL rifampicin, and 50 mg/mL kanamycin) for 48 h. Colonies were inoculated into LB medium (50 mg/mL rifampicin, and 50 mg/mL kanamycin) and grown at 28°C overnight. We collected bacterial bodies and diluted to a concentration equivalent to an OD_600_ of 0.8 and infiltrated into *Nicotiana benthamiana* leaves (4–6 weeks, with a 16-h light cycle at room temperature) using a 1-mL syringe. After three days of injection, we collected the injected leaves, and soaked them in 100 μM (+)-borneol, (−)-borneol, or DL-isoborneol for 12 h, respectively. Finally, the leaves were washed with ultrapure water, and stored in −80°C refrigerator after liquid nitrogen treatment.

We took 0.2 g samples and added 1.5-mL hexane to extract secondary metabolites. The supernatants were filtered through 0.22-μm organic filters and analyzed by GC–MS using Thermo TSQ9000 + trace1310. Helium was used as the carrier gas (1.2 mL/min) and then separated on the Agilent DB-5MS UI column (30 m × 0.25 mm × 0.25 μm). The GC oven temperature was programmed at an initial temperature of 50°C for 5 min, and then at 5°C/min from 50 to 115°C, increased to 295°C at 15°C/min, and hold at 295°Cfor 10 min. NIST20 Mass Spectral Library was used for target substance identification. All samples were performed for three biological replicates, and all data processing was performed with Xcalibur (Thermo Fisher Scientific Inc) and R packages.

### Resequencing and variant calling

DNAs from 39 leaf tissues, which contained 20 samples of WVV (10 from Yangchun, Guangdong and 10 from Jinghong, Yunnan), 10 of WVX, and 9 of WL, were used to construct the 150-bp paired-end libraries in BENAGEN (Wuhan, China), which were then were sequenced with the DNBSEQ-T7 platform (MGI, China).

The resequencing data from the 39 accessions initial quality control was performed by FastQC [85], and adapters were removed by Trimmomatic [86]. Then, BWA-MEM was used to map the treated data to the WVV genome [87]. Using Samtools, the mapped reads were sorted to generate bam files [88]. Then, Picard was used to remove duplicates, and GATK (v4.0.12) HaplotypeCaller was used to produce individual gvcf files [89, 90]. Finally, we used GATK CombineGVCFs to combine the gvcf files to obtain raw tcf files, and GATK VariantFiltration (QD < 2.0 || QUAL <30.0 || MQ < 40.0 || FS > 60.0 || SOR > 3.0 || MQRankSum < −12.5 || ReadPosRankSum < −8.0) was used to hard filter the SNPs. Only biallelic SNPs were selected for further analysis.

### Population genetic diversity and structure analysis

To infer the basal group of AF-origin plants, we constructed a phylogenetic tree based on 160 967 filtering SNPs (MAF ≥ 0.05, missing rate ≤ 0.1 and minimum distance between two SNPs ≥1 kb). We used VCF2Dis (https://github.com/BGI-shenzhen/VCF2Dis) to calculate the p-distance matrix of the 39 accessions, and the matrix was used to build the NJ tree. PCA was performed with plink [91], and STRUCTURE software [92] was used to analyze the population structure, with the likelihood of ancestral kinship (*K*) from 3 and 4, with both of the used SNPs filtered.

VCFtools [93] was used to determine nucleotide diversity (θ_π_) for the WVV-YC, WVV-JH, WVX, and WL populations with the following parameters: 100-kb sliding window and 50-kb step size. We used the same method to calculate the genetic differentiation (*F*_ST_) among different groups.

### Detection of sweeps

To avoid bias due to potential gene flow between WVV-JH and WVX, we conducted selective sweep analysis excluding admixed samples as identified by the structure analysis. We calculated the XPEHH [94], *F*_ST_, and π value of each SNP. The *F*_ST_ and π value were based on a sliding window of 10 kb and a step size of 1 kb. Regions ranked in the top 5% of the score in any of the methods were defined as putative selective sweeps.

## Supplementary Material

Web_Material_uhad128

## Data Availability

The data presented in the study were deposited in National Center for Biotechnology Information (NCBI), and BioProject accession number was PRJNA910288.
