# Supplementary material for: Multiomics comparison among populations of three plant sources of Amomi Fructus
Source: Hortic Res. 2023 Aug 1;10(8):uhad128. doi: 10.1093/hr/uhad128 (PMC10407604; doi:10.1093/hr/uhad128)
Supplement: Web_Material_uhad128 [file web_material_uhad128.zip › Supplementary Data 1-WVV.docx]

Amino acid sequences of BDH used in this study:

>Ro2635g23

MSCNTAVSRRLEGKVAIVTGGASGIGASTVRLFHDHGAKVVIADIQDDLGQTLADRLGRNISYTHCDVTDEDQVRALVDAAVAKHGGVDIMFSNAGIVEGPNSIFDVDKDELERLMGINLVGAFLAAKHAARVMVPAKKGCIIFTASACTEIAGIAGHSYTASKYGIVGLMKSLAVELGSHGIRANCVSPFGVLTGIVPDDEASKLMFEGIMSKVGNLKGKILTAEDVAMTVLYLASEEASYVSGVNLLVDGGYTVVNPTFINVITAGQS

>Ro350753g2

MSMKHQNLLYFLSMLQFVSQIPILKILPQPSFSFGPCATPLPNRQQPPPSPPSNPAATIGPQCERPLTPNTCSSSGRILQLRHLSAVAAINTPTGTDPSAAATIAPIEIVKYGYRHCKCRVTTVSPFKMILGGEFKIYHFVQFVCILSRLSGRVSLVTGGSSGIGESIVLLFRKHGAKVCIADLQDEQGQRLCDTLGGSSDVAFCHCDVTIEDDVKRAVDFTVDKFGTLDIMVNNAGVLGPPCPDIRDFELSAFDRVFDINVKGVFIGMKHAACIMIPAKKGSIISICSVSSTLGGIGPHAYTGSKHAVLVHLPEAERTEDSWDGFRRFVASNANLQGVELTAEDVANAVVFLASDEARYVSGMNLMVDGGFTSTNHALQVFR

>Ro378907g2

MPAQVIPELLFTGTQFTGMECISIPAIRRLEGKVAIVTGAAGGIGEATVRLFAAHGAKVIIADVDDNLGKSVADSLPAAQATYVRCDVSSEKEIETLINWTVSKYGKIDILFNNAGILGDQSRHKSILDFDAGEFDQIMTVNVRGAALGMKHAARAMIHGGGGGCVISTASVAGVMGGLGPHSYTASKHAIVGLTKNAACELGRYGIRVNCISPFGVATRMLVDAWREGGVGGGGVTEEEVEKMEEFVRCLANLKGETLRAKDVAEAALFLASDESRYISGHNLVVDGGVTTSKNCVGL

>Ro2635g25

MATFSTITALTRRLQGKVALVTGGATGIGECTAKLFSIHGAKVAIADVQDGLGQSVVKQIGASNSTYIHCDVTNEDHIRNAVDETVSVYGKLDIMVNNAGIADPPKPRIADNEKADFERVLAVNVTGVFLGMKHAARVMIPARRGAIISMASLSSGIGGAATHAYATSKHAVLGLTRNLAVELGQFGIRVNCLSPYACATDLSRKYLELDDEALEQAMSSMANLKGRTLKTADVANAALFLASDDAQYVSGQNLFIDGGFGIVNSAMQIFNYPES

>Ro1267g196

MLRLLTRSDPKNQIGNVVWASIRTNWFSSTAPVAVAGRRLEGKVALITGGASGLGKATAHAFIQQGAQVVIADINTQLGPRASLELGPQAQFMACDVAIEAQVSDAVDLAVARHGKLDIMCNIAGIAGSPLPPSIVDLELDEFDRVMAVNVRGTMSGIKHAARVMIPACSGSILCTASISGLLGGLGPHPYTVSKFAIPGIVKSLASELCGHGVRINCISPSPIPTPLVIEQFYKIVPNASRAEIVGMINGLGELKGAICEEEDVAGAALYLASDEAKFVTGHNLVVDGGFTSFKNLNFP

KLS

>Ro393439g4

MDSDESKFDMTNHLPSKRRLQGKVAVITGGARGIGAATAKAFAKNGANVVIADILDEPGAELAAAIGGQYVHCDVSVEQDVERAIQVAVDWKGRLDIMFNNAGICGPEGSITSLKMNQLAALLEINLNGVVHGIKHAARAMIEGKNAGTIICSASSAATMGGLASHAYTLSKAAILGVVRSSACELGLHGIRVNSVSPHGVPSEMLMPAYRRFLGNQNLQPQDVSKIVGEKGSLLRGRGGRMKDVAEAAVFLASDEAGFITGHNLVIDGGYTCASNQMSFIYQGQG

>Ro2635g22

MKMKPESNGFHTSKRLEGKVAIITGGASGFGEATAALFVRHGAKVVIADVQDDRGSALCRDLGLPNQISYVHCDVTSDADVSAAVDLAVSKYGGLDIMFNNAGIPGGLDFTIVDADNDNFRRVFEVNVYGAFLGAKHAARAMIPARRGGAILFTASVASAVAGESPHSYAASKHAVVGLMRNLCVELGQHGIRVNAISPCAVATPLLTGAMGVEKAVVEDIICASANLKGVVPTAEDVAEAALYLGSDESKFVSGLNLVVDGGYSTTNQSYSRVITSVFAPKSPS

>Ro372002g2

MNSFLMLFARKKKIGVKCLKLSGRVSLVTGGSSGIGESIVLLFRKYGAKVCIADLQDEQGQRLCDTLGGSSDVAFCHCDVTIEDDVKRAACGGLHCRQVRHPRHNGCFDWDEARCSHNDSGQERVDNINLQRVEHLGRHRASCVHGSKHAVLGLTKNVAAELGKHGIRVNCVSPYAVATSLALAHLPEAERTEDSWDGFRRFVASKANLQGVELTAEDVANAVVFLASDEARYVSGMNLMVDGGFTSTNHTLQVFRTKPSAPAEFYKSGDPYLPPPTIPLLLVICNPSQLLSYSPRVSAPESRYILFYFKISLSRMGLWTLLEGCLLLANALAILNEDRFLAPRGWSFQEYSGIKRNSFKGQILGLIYATQYLRVPLILLNLLCIIVKMVSG

>Ro1470g122

MSKPRLEGKVALITGAASGIGEEAARLFVKHGAAVVVADIQDELARQVIASMNSDRVSYRRCDVRDEEQVAAAVSFTIEKYGGLDVLFSNAGTLGPIASILDLDIQAMDNVLATNVRGVAATIKHAARAMVERNVRGSIICTASVAACIGGSGPHSYSAAKCAVVGLARSACGELGKHGIRVNCISPFGVATPMVCAAYGVTPGDIEANSSSAANLKGIVLKTKHIAEAALFLASDESAYVSGQNLAVDGGFAVVNQSYSSSSF

>Ro1051g326

MMGICNICGQEYVHKAKTDMAHIDNVSIRSTLCPMIRVEGKVASSPAPSGHRLDELRQGELPPLRCQKCRAGGGHRKLRRREIRRPRHPLQQRRNPRPNRRHIQAMDNILVTNVRGVAGTMRHAARAMVKRNTKVSIICTASVASCIGEAARLFGCEMRGTYGATPGVIEGNSCVVANLKRIVLKAKHIAKAALFVASDESAYISGQNLAVDGGFTAVNHSYSFSSF

>Ro1272g28

MANNLLKKKLEGKVAIVTGGASGIGETTARAFADHGARAVVIADIQPEKGRAVAESIGPQRCSYVHCDVTDEEQVAAMIEWTATTYGGLDIMFSNAGLSSSSLQTVLEFDLSQFDRVMRVNTRGAAVCVKQAARKMVELGTRGAIICTASSAAARGGANLTDYVMSKHAVLGLMRSASMQLGAHGIRVNSVSPAALLTPMTAKVGLTTAADVESAFGPVTSLKGVALTVEHVAEAVVFLASDEGAFVSGHDLAVDGGLVSLPFNVVTQ

>Ro1470g21

MPAAQLLPETLSQAVHGKETAAPSFKRLEGKIGIVTGGARGIGEETARLFAAHGARVVIADVEDALGNSVADSLSPHATYVHCDVASEADIETLINSTVAAHGKLDVLFSNAGILGDQARRKSILDFDAAEFDRVMVVNARAAALGMKHAARAMIRGGGGGCIICTASVAAVVGGMGPHAYTASKHAVVGLVKNAACELGKYGIRVNCISPFGVATSMLVNAWREEEEGGGEVSEVEMEKMEEFVRGMANLKGATLRTRDVAEAAVFLASDESKYISGHNLVVDGGVTTSKNCVGL

>Ro2006g59

MATCTAIVKSPQSLPLRLSGRVALVTGGSSGIGESIVLLFRKHGAKVCIADLQDEQGQHLCETLGGSSDVAFCHCDVTIEDDVKRAVDFTVDKFGTLDIMVNNAGVSGPPCLDIRDFELSAFDRVFDVNVRGVFIGMKHAARIMIPAKRGSIISISSVSSTLGGLGPHAYTGSKHAVLGLTKNVAAELGKHGIRVNCVSPYAVATSLALAHLPEAERTEDSWDGFRRFVAENANLQGVELTAEDVANAVVFLASDEARYVSGMNLMVDGGFTSTNHALQVFR

>Ro1051g325

MSKPRLEGKVALITGAASGIGEEAARLFVEHGAAVVVADIQDELAYQVIASMNSDKVSYRRCDVRDEEQVAAAVSFTIEKYGSLDVLFSNAGTLGPIASILDLDIQAMDNVLATNVRGVAATIKHAARAMVERNVRGSIICTASVAACIGGSGPHAYSAAKCAVVGLARSACGELGKHGIRVNCISPFGVATPMVCAAYGATPSQIEENSCATANLKGIVLKTKHIAEAVLFLASDESAYVSGQNLAVDGGFAAVNHSYSSGSF

>Ro2635g18

MAKRLEGKVAVITGGASGIGECAAKLFVRHGAKVIVADVQDDLGRAVCRNIGSHEVISYVHCDVAIEADVMGAVDFAVSEYGKLDIMFSNAAVTGKNNSRILAAEYEDVKRVFDVNVFGAFMCAKHAARVMIPAAKGSIVFTSSVASVTHGSVAHAYVASKHALVGLTKNLCIEMGAYGIRVNCVSPFGVPTPMLMAALRMTEKVEVEEFVSEIANLKGEVVGVDDVAEAALFLVSDEAK

YISGQNIVVDGGYSLTNIALAESVKKLNISS

>Ro371090g1

MSKPRLEGKVALITGAASGIGEEAARLFVKHGAAVVVADIQDELARQVIASMNSDKVSYRRCDVRDEEQVAAAVSFTIEKYGGLGVLFSNAGTLGPIASILDLDIQAMDNVLATNVRGVAATIKHTARAMVERNVRGSIICTASVAACIGGSGPHAYSAAKCAVVGLARSACGELGKHGIRVNCISPFGVATPMVCAAYGVTPGDIEANSSSAANLKGIVLKTKHIAEAALFLASDESAYVSGQNLAVDGGFAVVNQSYSSSSF

>Ro1111g42

MKRVCKSKQLAIYGTIYLQKGKVAVITGGARGIGAATAKAFAENGATVIIADILDEPGAELAAAIGGQYVHCDISMEQDVERAIQAAVNWKGRLDIMFNKAGISGPQGSITNLKMEQLAALLEINLNGVVHGIKHAARAMIGGKTQEQSYVHRVQQPRWEV

>Ro1153g949

MASTSLRRLEGKVALITGAASGIGEAAARLFSRHGAKVVIADIQDELARNVCKDLGPPSSASFVRCDVTKESDVETVVNTAVSTYGRLDIMINNAGIVEGPGQINILDCQLSEFKRVVDVNLVGAFLGTKHAARVMIPRRSGTIITTASVGSVIGGASSHAYTSSKHGVVGLMRNVAVELGGHGVRVNCVSPYILATPLSRGFMKMDDDGIGGCYSNLKGVVLMPEDVAEAALYLASEESKYVSGHNLVVDGGFSIMNQGLKIFDLIDS

>Aa1G03968661

MFLLVYVCLLSILMFNFCYNLCRLEGKVALITGAASGIGECTAKLFAEHGAKIIIADIQDQLGQAVSEAIGSSDSIYAHCDVTKEEDVINAIDIAVATYGKLDIMLNNAGIMDAYKARVIDNEKTDFERVLSVNVTGAFLGMKHAARVMVPARGGSIISMASIASNIGGMATHAYTCAKHAMVGLTKNLAVELGQFGIRVNCLSPYAVATPLATSYLGLDAEALEKKMHSIGNLKGVMLKADDVAKAALFLASDEAKYISGHNLFIDGGISIVNPSFSMFQYPDNLS

>Aa1G03968801

MAATTKRLEGKVALITGAASSVGECTAKLFAEHGAKIVIADIQDQLGQGVCETIGSSNSIYVHCDVTKEEDVQNAVDIAYATYGKLDIMFNNAGILDPYKARVIDNEKTDFERVLSVNVIGVFLGMKHAARVMVPTQGGSIISMASTASIMGGVATHAYTCAKHAVVGLTKDLAVELGQFGIRVNCLSPHAMATPMATNFLGLYGQDFENKVNLSANLKGVTLTIDDVAKAALFLASDDAKYISGQNLFIDGGFGIVNPSFNMFQYLDNL

>Aa1G03968851

MAATTKRLEGKVALITGAASGVGECTAKLFAAHGAKIVIADIQDQLGQGVCENIGSSNSIYVHCDVTKEEDVKNAVDIAYATYGKLDIMINNAGIMDPYKARVIDNDKTDFERVLSVNVTGVFLGMKHAARVMVPARGGSIISMASTGSIVGGVTTHAYTCAKHAVVGLTKNLAVELGQFGIRVNCLSPSAMATPMATNFLGLYGEDFENKVSSTANLKGVTLTIDDVAKAALFIASDDAKYISGQNLCIDGGFGIVNPSFNMFQYPDNI

>Aa1G03968861

MFHSIHIPSSCSCKKMTSSTRRLEGKVALITGAASGIGECTAKLFAEHGAKIVIVDIQEQLGQAVCEAIGSSNAIYVHCDVTNEEDVKNAVDVAVATYGKLDIMFCNAGIADPNKPRIVDNEKADFERVLSINVTGVFLSMKHAARVMVPAKSGSIISTASLASNIGGAASHAYCCAKHAVAGLTKNLAVELGQFGIRVNCLSPYAMVTPLATSFVELEGEALENVMNSRANLKGVTLKTDDVAKAALFLVSDEAKYISGQNLFIDGGLSIVNPSFNMFNYPENA

>Aa1G03991021

MSQSINPLYKTLGFYICCTSSISLSCYLLLSETMATVIKRLEGKVALITGAAVGLGECTAKLFAKHGAKVIVADIQDQLGQDVCEAIGSSNSKFVHCDVSKEADVKNAVDVAVATYGKLDIVFNNAAILDPYKLNVVDIEKSDFERLLSINLTGVFLGMKHAARVMVPRQSGSIISTCSVATQLSGMSSHAYTASKYAVVGLTKNMAVELGQFGIRVNCLSPSGMFTPMVKHSLNVTQEDFEKTVSSSGILKGVTLKADDVADSALFLVSDDAKYITGQNLIVDGGHTIASPINIFATHSHKS

>Aa2G01123901

MLRSIKPTCISISKGVVASTFQIERFSSHAQSKLEGKVALITGAASGIGKETATKFINHGAKVVVADIHNELGHDTATQLGPNASFISCNVTNESEIANAVDFTVSKFGQLDIMYNNAGIPCMTPMSIVDLDLNRFDQVMSVNVRGILAGIKHASRVMIPRESGSILCTASVTGILGGLAQHTYSVSKFVVIGIVKSLAAELSQHGVRINCISPFAIPTSFVMDEMKGYFPNLKDEDIMKMVRDAGGFKGNYCEPSDVANAAVYLASDDAKFVNGHNLVVDGGFTSHKGIKFSIPDTEH

>Aa3G02962381

MSQSINPLYKTLGFCICCKPSIPLSSYLLLSETMATVTKRLEGKVALITGAAGGLGECTAKLFAKHGAKVIVADIQDQLGQDVCEAIGSSNSKFVHCDVSKEEDVKNAVDVAVATYGKLDIVFNNAAILDPYKSNVVDIEKSDFDRLLSINLTGVFLGMKHAARVMVPRQSGSIISTSSIATQLSGMSSHAYTASKYAVVGLTKNMAVELGQFGIRVNCLSPSGMFTPMVKQSLNVTQEDFEKTVSSSGILKGVALKADDVAQSALFLVSDDAKYITGQNLIVDGGHTIASPINIFATHSQKS

>Aa4G01855641

MASLTPKARLENKVAIVTGGARGIGECIVRLFVKHGAKVVIADVNDDLGKLLCQDLGSKFACFVHCDVTIESDIENLINTTIAKHGQLDIMVNNAGTVDEPKLSILDNEKSDFDRVVSINLAGVFLGTKHAARVMIPKCSGSIITTASICSVTGGVASHAYTSSKHGVVGLAKNAAAELGKYNIRVNCVSPYFVPTKLAFKFLNMDETSSFYSNLQGKTLGPQDIANATLFLASDESGYVSGHNLVVDGGYSVLNPAFGLFSWKP

>Aa4G01856281

MASSTPKARLENKVAIVTGGARGIGECIVRLFAEHGAKVVIADVNDDLGKLLCQDLGSKFACFVHCDVTIESDIENLINTTIAKHGQLDIMVNNAGTVDEPKLSILDNEKSDFDRVVSINLAGVFLGTKHAARVMIPKCSGSIITTASICSVTGGVASHAYTSSKHGVVGLAKNAAAELGKYNIRVNCVSPYFVPTKLAFKFLNMDETSSFYSNLQGKTLGPQDIANATLFLASDESGYVSGHNLVVDGGYSVLNPAFGLFSWKP

>Aa4G02064461

MTTTSSNDSPLVAQRLLGKVALVTGGATGIGESIVRLFHKHGAKVCIVDIDDQLGQQLCQTLGENTCFIHCDVTIEDDVSRAVDFSVTSFGTLDIMVNNAGIGGPPCPDIREFSLSTFDKVFDVNVKGPFMGMKHAARVMIPLKKGSIISLSSVASAIGGLGPHAYTGSKHAVLGLTKSVAAELGNHGIRVNCVSPYAVLTNLALAHLHEDERTDDAMAGFRAFIGKNANLQGVDLLREDVANAVLFLASDEARYVSGANLFVDGGFTCTNHSLRVFR

>Aa5G03368521

MAAVTTDALKLSGKISIITGGASGIGEATARLFASNGAFIVIADIQDELGQKVSNSIGPQHCTYFHCDISSEDDVISLINFTVEKYGRLDIMFSNAGIVSTCKQTVLDLDLTQFDKLFAINTRGTAACVKHAARAMVNQRVKGSIICTTSVAASKGASMRTDYVMSKHAVLGLVRSASNQLGVYGIRVNCVSPSAVVTGLSNRSPEEIEKIMKVYEGLSSLKGVRLSVEDVAEAVVFLASDGSKFVTGHELVVDGGLTKLPDEDDVKMYNCCCI

>Aa5G03368691

MAEVSTHALKLAGKISIVTGGASGIGEATARLFASNGAFVVIADIQDELGQKVSNSIGPQHCTYFHCDISSEDDVISLINFTIEKYGCLDITFSNAGIVSTCKQTVLDLDLTQFDKLFAINTRGTAACVKHAARAMVNHHVKGSIICTTSVVASKGASMQTDYVRSKHAVLGLVRSASKQLGVYGIRVNCVSPSAVVTGISSMSPEEIEKTMKVCEGLSSLKGARLSVEDVAEAVVFLASDGSKFVTGHELVVDGGLTKLPDEDDLKMYNC

>Aa5G03368941

MVNHYVKGSIICTTSVVASKGASMQTDYVMSKHAVLGLVRSASKQLGVYGIRVNCVSPSAVVTGISSMSPEEIEKTMKVCEGLSSLKGARLSVEDVAEAVVFLASDGSKFVTGHELVVDGGLTKLPDEDDLKMYNC

>Aa5G03369091

MAQVTALSILKLAGKISIITGGASGIGEATARLFATNGAFIVLADIQDELGQKVAESIGSQHCTYIHCDISNENDVISVINFTVKTYGRLNIMFSNAGIISTSNQTVLDLDLAQFDKLFAINARGTVACVKHAARAMVEHNIRGVIICTTSVGASKGASTRTDYAMSKHPVLGLVRSASKQLGVYGIRVNCVSPSFVVTPLLNRYEETKKRKKVYEGLTSLKGVELRVEDVAQAVVFLASDASSFITGHDLVVDGGLTKLPDADDFV

>Aa6G00256831

MLYKGSTMELCDHTHDGSQKLGTKRLTGKVAIVTGGARGIGGATAILMAENGAHVIVADVLDEVGANLANSIKGLYIHCDVSKESDVESAVQLALSWKGKLDILFNNAGILDSGRSITNLEMKKLSTLINVNINGVIHGIKHAARAMISVGNKGSIICSSSSAAIMGGLASHSYTLTKGAILGISRSAACELGVHGIRVNCVSPHGIPSEMLMKAYREHLGKSDMTIEEVSNIISEEGSLLHGKCGSFEDVAQAVLFLASDESGFITGHNLVVDGGYTSASVLMSFIYRDKMI

>Aa6G00406241

MNGVYPHRMLEGKVAIITGGASGFGESTVRLFAKHGAKVVIADIQDQLGLSLCNDLVNKIGDNVIYLHCDVTKESDIENTVNTAVSKFGKLDIMFNNAGIPGNLDFTILNSDNENFKRVFDVNVFGSFLGAKHAARVMIPAKRGVILFTSSVASVLAGESPHSYTVSKHAVIGLMKNLCVELGQHGIRVNCISPGSVSTPLVTTAMGVDKEVVDGILCASAVLKGVVPTADDVAEAALYLGSDASRYVTGVNLVVDGGYSTTNPTYSRVIKQTFEDLAKKNEGCNGNGVSHAT

>Aa6G00467811

MADVSALPTLKLAGKIAIITGGASGIGEATARLFAANGAFVVIADIQDELGENVANSIGKQHCAYFHSDITDEQQVIALVDFTVKTYGRLDIMFSNAGIVSNSDQTVLDLNLTLFDKLFAINARGTAVCVKHAARAMVEQGIRGCIICTASAAASKGYSMHTDYIMSKHAVLGLVRSASKQLGAYGIRVNCVSPSAVVTPLLSKIGVSLPEDTKKMMNAYKGLSSLKGIELSVDHVAEAVLFLASNSSSFITGHDLAVDGGLMKFPDADDFVRYNP

>Aa6G00569111

MDLLAHNSLKSMELDHKDGDAHKLATKRLINKVAVVTGGSRGIGGATAKLLAEHGAHVIIADVLDELGTSLANSINGLFVHCDVSVESDVEAAIQLAITWKGKLDILFNNAGIGDIGGSITTLDMKRVSKLVEVNINGVVHGIKHAARAMILGGNGGSIISSSSTAAIMGGLGSHAYTLTKEAILGVTRSSSCELGTYGIRVNCVLPHAVLSDMLVDAYRSFKQDVTTEEIRQTVSENGSLLRGRCGMVEDVAEAVLFLASEQSGFITGHNLVIDGGYTSSSINMTFIYREKKS

>Aa7G04674971

MANATVDVPITIPRLAGKVALITGGARGIGESIARLFTKHGAKVVTADILDELGQTAVENIGLEKASYIHCDVCIESDIEKAINYTIDKHGKLDIMVNNAAIAGDLKFSILDNNVSDFERVLSINVTGVFLGTKHAARVMIPARAGSIISIGSIASTVGGNCSHAYTSSKHAVAGLTKNVAAELGEFGIRVNCVSPHYLVTPATVPIVKKYPDLYTNVYSNLKGIDLKEQDVAEATLFLASDESKYISGHNLAIDGGFTVINPSFGLFSKGKAT

>Aa7G04675101

MCIELDIENAINFAVKEHGKLDIMINNAAITGQPKLSILENEKSVFERVMSVNVTGVFLGTKHAARVMILACSGSIISIGSVSSTIGGVADHAYTCSKYAIVGLTKNVAAELGVFGIRDNCMSPYFIATSMGTSVSKNHPDLFSNAYSNLKGMVLQVDDVAHATLKLYKQGWSKKLGIKGAEIFFLKLTNKAIGCVGGGRAPPGSPLSPSLYISGHNLALDGGFTTINPSFGLFSRGKFT

>Tc01G1340

NPVFSSAQALDYFLCNHSRAGKSCFNLTTMFRRQFLSRLCNAVAGTGHESIRFSSSEAGKRLEGKVALITGASSGIGKATATEFVRHGAKVVVADVNDELGKAAAGQLGSSADFVHCDVRQESQVSDAVDYTVSKHGQLDIMYSNAGVPGPIVAGIADVDMADFDHVMNINVRGAFLGMKHAARAMVPRKQGCILCTASIAGLIGGVAPHPYAISKFAIPGLVKSVASELTRHGIRVNCISPYAIATPFALEGLRELYPGRSDDDLATYLDQCGELKVTGVRCEVKDIAKAALYLASEDGRYITGHNLVVDGGFSVIKQFLMPTP

>Tc05G1528

NMSNGEAVQGRLEGKVAVITGGAAGIGEATVRLFTKHGAKVIIADIADEAGLKLSESVSPLGTFIHCDVSKEEDVSAAVDLAVEKHGKLDIMFNNAGTISSEIRSAVEYDMKKFDRVMNINVKGVMHGIKHAARVMIPHKKGCIINTASIAGLLGGVAPYSYTASKHAVIGLTKNGAAELGKFGIRVNSVSPSGLATYFSMQHYEEITEERKTEVEGFFHSRANLKGATLKVEDMAQAALFLAGDDSKYVSGHNLVLDGGSSV

>Tc06G0545

MKRNLAFNLLRGIWNRGCSTNIPQIQPKRMEGKTALITGAATGIGEATARLFAAHGARVVVADVQEAAGSKLAAELGNGSEFVRCDVRSEEDVAAAVDRAVAAAEKRVLDVFHYNAGVLGALGPIDEMRMEDFDYTMSVNLRGAVLGVKHAARVMKPAKGGAILCTGSVASVLGGMGPHGYAVSKTAMVGLVRSAAVELRRFGIRVNMVSPDSVATPILGRALEKLELAADSSLAAAEQFVADTSLMGGRSLTTLDLARAALFLASEESGYVSGHNLVVDCGNTVTKPNDTALWYTVTMPAEN

>Tc09G2102

MRRLEGQVAIVTGGSNGIGAATARKFVAEGAYVASFVMCDVAVESHVEAAVNRAVEEKGQLDIMLNNAGMMHPRGHAIAHVQLDTWERVMAVNVTGSMLGMKHAARVMMPRRRGCILLNCSVLGLVKTDYASYGYVTAKHAVVGLMKSGAVELGKVGIRVNAVSSNAIVTAMIEKWLDEVSDGACPKEVFEEDMARCATFTGKRLTVDDVANAFMFLASNEASYINGHNLIVDGGYSVHGRNIVDFKSPSSLD

>Tc10G1292

LLEGKVAIITGGASGIGAATVRLFTKHGAKVIIADIADEPGKKVAHSVSPPATYFHCDVTNEKDVRAVVNLAIENHGRLDIMLNIAGTIDTYKGSVAEYEMEEYERVTKVDVKGVLHGIKHAARVMIPNQKGCIISVASIAGILGGTSPYAYTAAKHAVIGLTKNGAAELGKYGIRVNCISPYGVATPLFAQLALNEEYSPGPVSKEDKVKMEALANGIANLKGPTLEAEDIAEAALYLAGEKA

>Tc10G1445

SNNSIQGITKYMNYIFAKLLNLCDAKRRLEGKVAIITGGASGIGEATVVLFVRHGAKVIIADIAAEAGIKLAESLSPSATFIHCDVTKEQDVRAAVDFAIEKFGRLDIMLNNAGTTDIPEGGAAEYDMEEFERVMNVNVKGVMHGIKHAARVMIPNKKGCIISMGSIAGVLGGKGSFAYTASKHAIIGMTKNGAAELGNYGIRVNSVSPSAIPTNIGSQFLKYEMKAILEAISKKIAILKGVTLEVEDIAEATLYLASEESKYVSGHNLVVDGGYTV

>Tc10G1447

LQGKVALITGGSGGIGEATVRLFANHGAKVIIADIEDDAGIKVAHSLSHSATYTRCDVTKEKDVCAAVDLAMEKHGKLDILYNNAGLINIQKGSVAEYDMEQFQRVMNVNVKGVMHGIKHAARVMIPNKKGSIISTASIAGVLGGICPYSYTASKHAVIGLTKNGAAELGKYGIRVNSVSPGVVATDFALRYMGFIPSAEGKAELETSLSSISNLKEATLEAQDIAEAVLYLASEESKYVSGHNIVVDGGVTVVNKDLGLYK

>Tc10G1448

MYLTATRPNLMYVVSLISRFMASLTELHLQAAKRVLRYLKGTVDLGVFYRKEGNGELMAYTDNDYAGDVDDGKSTSGYVFLLSEGVVSWSSKKQRVVSLSTTEAKFVATASCACQGVWMRRVLEKLGHSQGKCTTMLCDNSSTIKLSKNPVMHGCNKHIDVSFHFLRDLTRDGVVEMKHCVTQEQVADIMTKPLKLDVFLKLRESMDIILNCFVLRVYNTPAYSAGQLHHFFPFVNLKKTMSTGEPAAALQGRLHGKVAIITGGAAGIGEATVRLFTKHGAKVIIADIADESGKKVAETLSPWTTYIHCDVSKEQDVSSAVDLAVEMHGHLDIMFNNAGRVESQKKLGAAEYDMEEFERVMNVNVKGVMHGIKHAARVMIPNKKGCIISTGSIAGTLGGTGPYGYTASKHAILGLTKNGAAELGKYGIRVNSVSPTGLATAFTLQYANEEDKAKLEAFCDSVANLKGPTLQVEDVAEAALYLASEESKYVSGHNLVVDGGFSVVNHEWGLYR

>Tc10G1457

MKNNLKIMKCRLQGKVALITGGSGGIGEAVVRLFANHGAKVVISDIADDAGIKLAQSIPPGATYIHCDVTNEKDVRTAVDLAVGKHGNLDIMYNNAGIIHTQRASAAEYEMEEFLRVMN

>Tc10G1466

MEEYERVMKVDVKGVLHGIKHAARVMVPNQKGCIISIASIAGVLGGTSPYAFTAAKNAVVGLTKKGAAELGKYGIRVNCISPYGVPTVLTVQLLLNAEYSPGPISKEDKLKVDAVTNGIANLKGVTLEAKDITEAALYLAGEKARIIIKDLDKNSIEECKNICREAHPR

>Tc10G1670

MSTPVVHGRLEGKIAIITGGAAGIGEATVRLFTKHGAKDVKAAVDFAMEKHGKLDVMFNNAGTGDSHRKSVADYEMEEFERVMKVNAKGVMHGIKHAARVMIPNRKGCIISTASVAGISGGLCAYAYTASKHAVIGLTKNGAAELGKYGIRVNCVSPSGVATELVMGFTRTHDPAEVEGFLNSTSNLKGVTLKAEDIAEAALYLASDESKYVSGHNLVVDGGFTVVNHNWGLY

>Tc10G1671

RTMSTPVVHGRLEGKIAIITGGAAGIGEATVRLFTKHGAKVIIADIADNEGQKVAESLSPLATYIHCDVSKEQDVKAAVDFAMEKHGKLDVMFNNAGTGDSHRKSVADYEMEEFDRVMKVNAKGVMHGIKHAARVMIPNRKGCIISTASVAGISGGLCAYAYTASKHAVIGLTKNGAPEFGKYGIRVNCVSPSGVATELVMGFARTHDPAEVGGFLNNTSNLKGVTLKAEDIAEAALYLASDESKY

>Tc10G1674

MFFHGFICIYVVDLASCADKGYLETVRKACEEIGCFRIINHGIAPDVLCNADLLCRDVFSLPTETNKKNVSPVPFASYVGGIPFLPFYESLAIDNPDQEAIKDFALLMWPHGNPNFWEGFPALFPSGILSADVFARYCRRRDYNVIYICATDEYGTATETKAMKENSTPKQICDSSSFSVTNLLNVPSSGHVSGYQMNSNTSSCGGIDESGVSQCVSNVTEMNPPTRTFTKLSMVMTVEEGEGEKNNQAERTMSTPVVHGRLEGKIAIITGGAAGIGEATVRLFTKHGAKVIIADIADNEGQKVAESLSPLATYIHCDVSKEQDVKAAVDFAMEKHGKLDVMFNNAGTGDSHRKSVADYEMEEFDRVMEVNAKGVMHGIKHEARVMIPNRKGCIISTASVAGISGGLCAYAYTASKHAVIGLTKNGAAELGKYGIRVNCVSPSGVATELVMGFTRTHDPAEVEGFLNGTSNLKGVTLKTEDIAEAALYLASDESKYVSGHNLVVDGGFTVVNHNWGLY

>Tc11G2588

LKGKVAIITGGARGIGEATVRLFAKHGANVVIADIEDTAGKILSDSLSPAVTFIHCDVRKEEDVAHVVNMAISRYGVLDIMFNNAGILGDQANRKSILDFDADEFDNVIAVNVKGVCLGMKHAARVMIPRRSGCIISTASVAGIMGGLGPHGYTASKHAIIGLTKSAACELGKYGIRVNSISPFGVATPMLRNAWRKKDGLEEEEERIEEFVRSLANLKGTTLRTEDIAEAALYLVSDEA

>Cc05G0442

MVNFYRLEGKVALITGGASGIGASTARLFICHGAKVIIADVQDELGHSICNEIGSDEYVHFIHCDVTKEEDICNAVDCAISKYGKLDIMFNNVGISGDKKPSLLDINKEDFEKVYNVNVFGAFLGAKHAARVMIPAKKGCTLFIASIAFVACTGRWHEYVSSKHAVVGLTKNLCVELGQFGIRVNCISPYVIVTPLTKNMFAMDESEIEKLMTSSATLKKVTLKVEDVAEAALYLASDESKYISGLNLVIDGGYCGTSRFPNVVIADDSAPEGGCAKNPFVLISESDPLSARPAAGVLKSSSPRRLLLGFLSSECIDQAVRYASVFGGVLLSLLFELFSISGCWGYLVLLLGFVASFTPPSVLSLSVMCLVVVASFPPPVFSLSVLCLVGVAPSLSLEGCCHADSI

>Cc11G1234

MSKPRLFDIYGPCIFYRLEGKVAIVTGGASGIGEVAARLFVNNGAFVVIADIQDELGHRVVNSIDPAKCCYRHCDVRDEKQVQEMVDHAIEKYGSLDIMFSNAGIVGSLTGILKLDLGDLDNTMAINVRGVAATVKHAARAMVARQIRGSIICTASVAACIGGSGPHAYTISKHALVGLVRSTASELGKHGIRVNCISPFGVATPLTCQLGNLDPSEVEANCCAMSNLKGTVLKPQNVAEAALFLASDESSFISGHNLVVDGGYSVVSHAFSSFQ

>Cc05G0443

MKLLDLYSGCGAMSTGLCLGANIAGVNLVKVRNEKVEDFLLLLKEWEKLCKHFSLLGNIKVQSRSSDAIFSEEDDEVDEDIEEEAKTPSGEFEVGKLLSICFGDPNEIGVQGLKFKEASTLTSNFIAGSAAIDVQQLALPEFKGLSGHGVLTRSSKHEDLMERQYDGRLLPPTDFETSASILNFERQNLREFVEQDGEEHNEPYVKTSVASNTPIFCTYSLGVNMSSNLQDPIARRLEGKVALITGGASGIGASTVRLFIRHGAKVIIADVQDELGHSICNEIGSDEYVHFIRCDVTKEEDICNAVDCATSKYGKLDIMFNNAGICGDMKPSLLDIGKEDFEKVYNVNVFGAFLGAKHAARVMIPAKKGCILFTASIASITSTGGWHAYVSSKHAVVGLTKNLCVELGQFGIRVNCISPYGVTTPLTKNVFAMDESEIEKLLTSSATLKEVTLKVEDVAEAALYLASDESKYISGLNLVIDGGYCGTSRVHDSASEGGRAKNV

>Cc07G1111

MALYDSRLEGKVAIITGAASGIGEATARLFAENGAFVVIADIQDELGQSVAASIGLDKSSYRHCDVRDENQVEETVTFTLEKYGRIDVLFSNAGIIGPPTSILDMNLEELDNTFAVNVRGVAATIKHVARAMVQHKTRGSIICTMSVAGIVGGTGPHAYSTSKSALIGLVRSTASELGKDGIRVNGVSPHGVATPLSCGLSGTEPSQVEAICCSTSCLKGVVLKTRHVAEAVLFLASDESLFISGHNLAVDGGFSVVNGGFVLYQ

>Cc02G2181

MSVQVMPEQTHQTLPVLGRDNPVSIQVIPEQTHQTIPVLGRDNTALSPKRLEGKVAIVTGGARGIGEATVRLFAKHGAKVVVADIEDMAGRALAASVGPSVTFVHCDVSLEEDVENLIDSTVSQHGRLDILFNNAGVLGNQSKRKSIIDFDADEFDRVMRVNVRGAALGMKHAARVMVPRGMGCIISTASVAGVMGGLGPHAYTASKHAIVGLTKNAACELGRAGIRVNCISPFGVATPMLVNAWRGGEEEEDDCIGLSIPSEQEVEKMEEIVRNLASLKGPTLRPIDIAEAALYLASDESKYVSGHNLVVDGGITSSRNCVGL

>Cc10G0305

MTIACRLEGKVALITGGASGIGEYTAKLFCLHGAKVVIADIQDELGHSVCKDLGTSVASFIHCDVTNETDVQNAVDYTITTHGKLDIMFNNAGISDPPKWSILENDVADFERVLRTNITGAFLGTKHAARVMIPAGRGSIISTASVSSVVGAAASHAYTCSKHAVAGLTKNASIELGKYGIRVNCVSPYVVPTPLSRGFVGLDDEVFDKAMRSCANLKGVSLEGEDVAQAVLYLGSNDSKYVSGHNLIIDGGFTVANPSFGMFK

>Cc05G0530

MESLEQSGQFARADHKGRLGEQLSVLILASSGQFARVDPREGKVALITEGASGIGASIVRLFICHGAKVIIVDVKDELGHSICNEIGSDECVHFIHCDVTKEEDIHDTVDCVVSKFAKIDIMFNTAAISGDKKSSILDVDKNDFEKIYNVIVFGAFLGAKYTARRRVDAYVSSKHAVVGLTRNLCVESGEFGIRVNCISPRGVVTPMAMKMLAMDECEFEKLMCCMETLKGVTLKTKDVAEAAIYVANDESKYISGLNLVIDGGYPTNRASNMAIASASPSQGDQTKNA

>Cc05G0444

MSSNLQCPTAKRLEGKVALITGGASGIGASIVRLFIRHGAKVIIADVQDELGHSICNEIGSDECVHFIHCDVTKEEDIRDTVDCVVSKFGKLDIMFNNAAISGDKKSSILDVDKNDFEKVYNVNVFGAFLGAKHAARVMIPVRKGCIIFTASIASVTCKGGWHAYVSSKHAVVGLTKNLCVELGEFGIRVNCISPRGVVTPMAMKMLAMDECEFEKFMCCTATLKGVTLKTEDVAEAALYLASDESKYISGLNLVIDGGYSATNRASDVAIASASPSEGDQTKNA

>Cc04G1590

MNRDCAFLNRLEGKVALITGGAKGIGEVTARVFHQHGAKVVIADIEDDLGHLVCKDFGKNSASYVHCDVSKEVDVKNAVDATVAKFGRLDIMFNNAGIVDPWKSNITNNDEADFEKVLGVNVKGVFLGTKHAARVMVPVKRGSIINNGSISSVIGGIASHAYVASKHAVLGLTKNAAAELGQYGIRVNCISSFVLASSLTTNFISKDENEIESWAGRLANLKGVVLTAEDLALAALYFGSDESGYVSGHNFVLDGGFSIVNPSFGLFKQNP

>Cc02G1258

MELEGKVAIITGGASGLGKASAQEFIQQGAKVMIADVNKNIGLQTAEELGPHACFIHCDVTIEHQVADAVDFALLKHGRLDIMYNNAGIPGPSVPPSIVDLDLHEFDRVMKVNVRGTIAGIKHAARVMIPASKGCILCTASISGIMGGLGPHPYTVSKFAIPGIVKSAASELCRHGVRINCISPFAVPTPLVLDQLSAFYPGVGPNRLSDMVGGLGTLGGTRCDEADVARAALYLASDEAKYVTGHNLVIDGGFTCFKRLDFPAPRKVLV

>Cc01G0882

MGSLNALSAIARRLEGKVALITGGASGIGECTARLFSQHGAKVIVADIQDELGRSVCKDLGPTTSSFIHCDVTKEPDIKNAVDIAVSKYGKLDIMFNNAGIVDDPKSRIIDNEKADFERVLSINVTGVFLGTKHAARVMVPARSGSIISTASICSYLGAVASHAYTASKHAVVGLTKNGAAELGQFGIRVNCVSPYALVTPLAAKNFVKTQGEDLEKAFSVPANLKGVHLKAEDVAQAVLYLGSDDSKYVSGHNLLIDGGFSVSNHCFRMFDYSFA

>Cc11G1091

MALFLQKALLRNTSRRSVLMLKDWNSSRERNKGRQLSTEAGSKLAGKVAVITGAASGIGRATASEFIRNGARVVIADVQRQLGEETAKELGPNAAFIPCDVTQESEISAAIDFAISEHGRLDIMYNNAGIPGSVVFSITDLDLSEFDKIMNINVRGVVAGIKHAARVMIPRRTGCILCTASITGIMGGLSPHAYSISKFATAGVVKSAASELCKYGIRVNCISPFAIPTPFSTDPMSVLYPGLDESGVVEIIQGAGVLKGANCEPVDIAKAAVYLASDDAKYVSGHNLVVDGGFTVFKSIQFPPPDQMK

>Cc08G1393

MILCRLEGKVAIITGGANGIGESTVRLFWTHGAKVVIGDIHDDRGQALAQELDSNAMYIHCDVTNEDDVRNMVDQTVEKFGKLDIMYNNAGIAETTAPKSILTIDKASFERVLKVNLVGGLLGTKQAARVMVPARKGCILFTGSATASIACTINHSYVASKCGVAGLARNVSAELGQYGVRVNCISPYAVATDMMKGLLPNGDGKQLEAFLSEAGNLKGGVLRVDDVAQAALFLASDEASYVSGLNLVVDGGFSVANPSVAMALSRLN

>Cc05G1625

MGSSFVLSLVSRRLEGKVALITGGASGIGSCTAKLFSQHGAKVVIADLNEEMGQAVCKELGPQVASFIHCDLTKEDNVQNAVDATVSKHGKLDIMFNNAGITDAFKPSIVESDKSDFERVHSTNVMGVFLGTKHAARVMIPARRGSIINTSSVASTMGGLGTYAYTSSKHAVVGLTRNAAIELGQFGIRVNCVGPYAVPTPMVSSVLNNMGEEEIAKKAGACSILKGVLLDAQDIAQAALYLGSDESRYVNGHNLMVDGGNTQTNAALLAAIPS

>Cc07G0476

MFMLRRCFLMLKHGRMDNTSRCLSTEAGRLAGKVALITGAASGIGKATATEFIRNGAKVIIADIQRQLGEEAATELGPDAAFIHCDVTQESDVSAAVDYAISKHGHLDIMYNNAGITGSVTNTVADLNLEEFDRVMSINVRGVITGIKHAARVMVPRRTGCILCTASITGILGGISHHSYSISKFTVAGIVKSTASELCRHGIRVNCISPAAVPTPMVLSGVSDFFPGMDATQIIEIMRGAGTLKGVDCDTIDIAKAALYLASDDGRYVSGHNLVVDGAFTSFKNMELPAPEKVMK

>Cc07G0251

MGSSFVLSLVSRRLEGKVAIITGGASGIGSCTAKLFSQHGAKVVIADLNEEMGQAVCKELGPQVASFIHCDVTKEDSVQNAVDETVSKHGKLDIMFNNAGIIDAYKPSIVESDKSDFERVLSTNVIGVFLGTKHAARVMIPARCGSIINTSSVASRMGGLAPHAYTSSKHAVVGLTRNAAIELGQFGIRVNSVGPYVVPTPLATSLFNQGKEEIAKKAEACSILRGVLLDAQDIAQAAIYLGSDESRYVNGQNLMVDGGNTQTNAALLPAWTS

>So02G00229

LEGKVALITGAASGIGAAAARLFSRHGAKVVIADIQDELARDVCKDLGPPSSATFVRCDVTEESDVETAVNMAVSMYGRLDIMINNAGTAGAPQIQILDCQLSEFKRVVHVNLVGAFLGTKHAARVMIPRRSGSIITTASVCSAVGGASSHAYTSSKHGVVGLARNAAVELGAHGVRVNCVSPYITPTPLSRDFVGMSNEGIQGCYSNLKGVVLTPEDVAEAALYLASDESNGIGAASARLFSRHGAKVVIADIQDELARNVCKDLGPPSSATFVHCDLTEESDVETAVNTAVSTYGRLDIMINNAGIAGDPGQIKISDCQLSEFKRVVHVNLVGAFHGTKHAARVMIPRRSGSIITTASVGSALGGASSHAYTSSKHGVVGMARNAAMELGAHGVRVNCVSPYILPTPLSKDFVKMDDDGIRGNMSRPVLLVCVLLILIITSQFEWRQQLVSDVDAIATPKESQKQQRIQQREESIKEKIILSQEKNIQSLNELVRSLKEQLRQCRGNNETSNGAFSS

>So02G00233

MIPRRSGSIITTASAASVRGGGAPHTYTTSKHGVVGLMRNAAVELGAHGVRVNCVSPYIMATRLTRDFMKMDDKGFRSCFSNLKGVILMPEDVAEAALYLASDESKYVSGHNLIVDGGYVLS

>So02G00234

MASISCFMRPRLEGKVALIITGAASSIGAAAARLLSRHGAKVVIADIQDELARNVCKDLGSPSSATFVRCDVTEESDVEAAVNTAVSTYGRLDIMINNAGIGGDPDQIRFATASCPNSSTSST

>So02G00235

MGSSLLLSTVAKRLEGKVALITGGAGGLGAMTAKLFHQHGAKVVIADVVDPPLGDAASFVHCDVTKESDIANAVDAAVSMHGRLDIMFNNAGIMGRVDRDILDCDPADFDRVLRINVAGVFLGTKHAARAMRFAGAGGAIVNTASVCGVVGGVAPYAYTASKHAVVGLTRSAAAELGRHGIRVNCVSPYVFPSAMSRFLLGRAEEDELEDVTLKVKGRRLDGEDVAQAVVYLASDESRCVSGHNLVLDGGFTVTNSDLNMFT

>So03G00168

MAKNLVRKKLEGKVAIVTGGASGIGETTARVFADQGARAVVIADIQPEKGRAVAESIGPQRCSYIQCDVTDEEQVAAMIEWTATTYGSVDIMFSNAGFMSDSGQSVLELDLAEWERVMRVNARGMAVCVKQAARKMVELGSRGAIICTASVAATRGAVGITDYVMSKHAVLGLMRSASMQLGAHGIRVNSVSPGAVLTPMGAKGGVATPADVERHIGPVTSLKGAALTAELVAEAVVFLASDEAAFVTGVDLAVDGGMICMPFNLTPSQ

>So03G02280

MSETLLNVADSQGGHTVLLRDSENSDSDRVVPKSSWLLGKVAVVTGGASGIGESIVRSFVRHGAKVCVADIQDDVGRRLVESLSHDGSDATYCRCDVAVDLAVGKYGSLDIMVNNAGVAGSFAPDIRDAEVSNLERVLDVNVKGVFAGMKHAARTMIPRRRGSIVSTCSIAGVRGEQKRPKNRTNRETYSKVKSPNHYFVVKMSNVAGNTENGPAHEVANKTPNLAPIETQVLEKGTSQNPNAALEQAMVAGANLKGVALTTDDVADAVVFLASDEANYMSGMNLVVDGGVTAVTNAFRDLEQLTQPTKTISWEKLNFIMYDNICFNEEMDLNNLRGFSVAKQNLVV

>So03G02438

MDSDESKDDMTHHLPSKRLSGKVAVITGGARGIGAATAKAFAENGANVVIADILDEPGAEVAAAIGGKYVHCDVSVEKDVERAIQVAVDWKGRLDIMFNNAGISGPEGSITNLKMEQLAALLEINLNGVVHGIKHAARAMIEGRNAGTIICSSSSAATMGGLASHAYTLSKAAILGVARSSACELGLHSIRVNCVSPHGVPSEMLMTAYRRFLGNENLQPQDVRKIVGEKGSLLRGRGGSMEDVAEAVVFLASDEAGFITGHNLVIDGGYTCASNQMNFIYQE

>So03G04119

MPAQVMPELLFSGTQFPGIECISIPSIRRLEGKVAIVTGGAGGIGEATVRLFAAHGAKVIIADVDDSLGNSLAESVPAGQATYVRCDVSSEKEIETLINWAVSKFGKIDVLFNNAGILGDQSRHKSILDFDAAEFDNVMTVNVRGAALGMKHAASAMIRGGGGGCVISTASVAGVMGGLGPHAYTASKHAIVGLTKNAACELGRYGIRVNCISPFGVATRMLVDAWREGGGEGGGGATEEEEEEEEEEAAALAGGNERDLRERWRRMWRWSAAICCRDFVRFRNRVREIQIESEREEERER

>So04G00504

MFRTPFRKWSGARLLLTPASQRWFSQDTIRLFSKLEGKVALITGAASGIGKETATKFINNGAKVVLADVQKDAGHHTARDLGPNAAFVTCDVTKESDVSDAVDFAVSKFGHLDIMYNNAGIACYTPPSIVDLDMASFDRVMAINVRGVVAGIKHGARVMIPRQRGVILCTASVTGLMGGLAQHTYSVSKSGVIGIVRSVASELCKHGIRVNCISPMAIPTPFVMDEIKKYYPEVDPQKLVKMVHDFSVLKGAACEPADIANAAVFLASDDAKFVSGHNLVVDGGFTSFKNLSLPTPDQLR

>So04G01269

MSIKQRLEGKVALITGGASGIGEAAARLFAEHGAAVVVGDIQDELGRRVIASMKSDRVSYRRCDVRDEEQVAAAVGYAVEKYGGLDILFSNAGSLGPIASILDLDMEALDRVLATNVRGVAATIKHAARSMVERKVRGSIICTASVAACIGGSGPPAYSAAKCAVVGLARSACGELGKHGIRVNCISPFGVATPMVCAAYAATPAQIEANSTAAANLKGIVLKTTHIAEAALFLASDDSAYVSGQNLAVDGGFAAVNNTYSSTSF

>So04G03055

MLIAGPAHGIRVNIVSSGAVRTPLAAKVGLVTAADVESYAGPFTSLKGAALTAENVAEAAAFLASDEAAFVTGVDLAVDGGMICMPFDVTHSQ

>So05G03115

MLRYLLTRSEPKKQIGNVVWASITTRFSSTAAPGQGRRLEGKVALITGGASGLGKATAHAFIQQGAQVVIADINAELGPRASLELGPQAQFVACDVAVEAHISNAVDLTVGRQGKLDIMCNIAGIAGSPFPPSIVDLELDEFDRVMAVNVRGTMAGIKHAARVMIPAGSGSILCAASISGLLGGLGPHPYTVSKFAIPGIVKSLASELCGHGVRINCISPSPIPTPLVVEQFCKIVPNATREEIVGLINSLGELRGAICEEEDVAGAAVYLASDEAKFVTGHNLVVDGGFTSFKNLNFPKLT

>So07G01494

MASGAANVESPQSLPLRLLGRVALVTGGSSGIGESIVLLFRKHGAKVCIADVQDNQGQRLCETLGGSSDIAFCHCDVTIEDDVKRAVDFTVDKFGTLDIMVNNAGVSGPPCPDIRDFELSAFDRVFDINVRGVFIGMKHAARIMIPAKKGSIISISSVASTMGGLGPHAYTGSKHAVLGLTKNVAAELGKHGIRVNCVSPYAVATSLALAHLPEAERTEDTWDDFRRFVADNANLQGVELTMEDVANAVVFLASDEARYVSGMNLMVDGGFTSTNHALQVFRP

>So07G03037.1

MASNFFKAHSLAFSIMAGFSTLSALTRRLEGKVALVTGGATGIGECTAKLFSQHGAKVAIADVRPELGQSVVEQIGASNSRYIQCDVTDEDQIRNAVDETVSAYGKLDIMINNAGIADPPKPRIADNEKADFERVLAVNVTGVFLGSKHAARVMIPARRGAIISMASLASGIGGGATHAYTSSKHAVVGLTRNLAVELGQFGIRVNSLSPYACATDLSKKYLELDDEALEEAMRSMANLKGATLKTADVANAALFLASDEAQYVSGQNLFIDGGFGIANSAMRIFDYSQS

>So07G03038

MHGEALLQTRRQSCSRRHPRRIRPIRRQTNRRLEFEIHPLRRHRRGSDPQRRRREGLRLRQAAGTADPPKPRIADNEKADFERVLAVNVTGVFLGMKHAARVMIPARRGAIISTARRRTLTRARSMRWWG

>So07G03039

MLGVDDEALEQAISSAANLKGTTLKAADAANATLFLASDEARYVSGQNLFIDGGFSIVNSAMQIFNYPHSN

>So07G03041

MKPESNGFHTAKRLEGKVAIITGGASGFGEATVALYARHGAKVVVADVQDERGHALCRDLALPEQVTYVHCDVTSDADVSAAVEVAVSKYGGLDIMFNNAGIPGGLDFAIAEADNDNFRRVFEVNVYGAFLGAKHAARAMIAGGARARGGAILFTASVASAVAGESPHSYAASKHAVVGLMRNLCVELGQHGIRVNAISPCAVATPLLTAAMGVEKAVVEDIICASANLKGVVPTAEDVAEAALYLGSDESKFVSGLNLVVDGGYSTTNQSYSRVIKSVFAPK

>So07G03042

MAKRLEGKVAIITGGASGIGECAARLFVRHGAKVVVADVQDELGRATCQSIGPPEVISYVHCDVAIEADVAGAVDFAVSEYGGLDIMFSNAAVPGKSEAGILAADYEDVKRVFDVNVFGAFACAKHAARVMIPAANGCIIFTSSVASVTHGAVPHAYVASKHALVGLTKNLCIEMGEHGIRVNCVSPFGVPTPMLMSALRIVEKAEAEEFVSKIANLKGEVVGVEDVAEAALFLASDEAKYISGQNIVIDGGYSLTNVALRESVKKMNISS

>So07G03427

MPAAQTLSPALHGIETAAPSFKRLDGKIAIVTGGARGIGEETVRLFAAHGASVVIADVEDALGASVAASLHPAATYVHCDVASEADIETLITSTVAAHGRLDVLFSNAGVLGDQARRKSILDFDAAEFDHVMAVNARAAALGMKHAARAMIRTAGGGSGGGGCIICMASVAAVVGGMGPHAYTASKHAVVGLVKNAACELGKYGIRVNCISPFGVATSMLVNAWREEGGGGGVSEVEVEKMEEFVRGMANLKGATLRKRDVAEAAVFLESDESKYISGHNLVVDGGVTTSTNCVGL

>CcBDH3

TGKSELSTSNNLLLPHQRLEGKVALVTGGASGIGESIASLFQQHGAKVCVVDLQDELGRQVCDSLGGDSNACYIHGDVTVEDDLRRAVEFTVEKFGTLDIMVNNAGISGTPGIDIRYADIGEFQRVFDVNCKAVFMGMKYAAQVMIPRGKGSIVSLASVASQVGGIGPHAYTASKHAVVGLTKNVAAELGLYGIRVNCVSPYAVPTSLAMPHLPEGEAKDDALEGFLAFAERNGNLKGARLMPKDVANAVLYLASDEAQYVSGLNLVVDGGFTAVNHALEVFTKK

>CcBDH2

MSSNLQDPIARRLEGKVALITGGASGIGASTVRLFIRHGAKVIIADVQDELGHSICNEIGSDEYVHFIRCDVTKEEDICNAVDCATSKYGKLDIMFNNAGICGDMKPSLLDIGKEDFEKVYNVNVFGAFLGAKHAARVMIPAKKGCILFTASIASITSTGGWHAYVSSKHAVVGLTKNLCVELGQFGIRVNCISPYGVTTPLTKNVFAMDESEIEKLLTSSATLKEVTLKVEDVAEAALYLASDESKYISGLNLVIDGGYCGTSRVHDSASEGGRAKNV

>CcBDH1

MGSSSALSLVARRLEGKVALITGGAGGIGSRTAKLFSQNGAKVVIADLNEEIGQAVCKELGPQGATFIHCDVTKEDNVQNAVDATVSKYGKLDIMFNNAGIIELPKPSIVETEKSDFERVLSTNVIGVFLGTKHAARVMIPARRGSIINTSSNASIMGGITPHAYTSSKHAVVGLTINAAIELGQFGVRVNCVAPHLVPTPMTTSLFKMGEEELAARAASCSILKGISLKAEDIAEAALFLGSDESRYVNGHNLRVDGGYTLTNPTLRDALTS

>SoBDH2

MATGAANVESPQSLPLRLLGRVALVTGGSSGIGESIVLLFRKHGAKVCIADVQDNQGQRLCETLGGSSDIAFCHCDVTIEDDVKRAVDFTVDKFGTLDIMVNNAGVSGPPCPDIRDFELSAFDRVFDINVRGVFIGMKHAARIMIPAKKGSIISISSVASTMGGLGPHAYTGSKHAVLGLTKNVAAELGKHGIRVNCVSPYAVATSLALAHLPEAERTEDTWDDFRRFVADNANLQGVELTMEDVANAVVFLASDEARYVSGMNLMVDGGFTSTNHALQVFRP

>SoBDH1

MNSSSAVSKRLEGKVAIVTGGASGIGASTVSLFHDHGAKVVIADIQDNLGQTLAGRLGRNISYIHCDVTDENQVRALVDATVAKHGGVDIMFSNAGIVEGPTVSIFDADKGALERLLGINLVGGFLAAKHAARVMSPTKKGCIIFTASACTEVAGISGPGYVASKYGIVGLMKSLAAELGSHGIRANCVSPFGVLTGIAAGDDKTKLMFEGLMSKVGNLKGKILTADDVAKAALYLASDEASYVSGVNLVLDGGYSVVNP

>LiBDH

MASTVLRRLEGKVALITGAASGIGESAARLFSRHGAKVVIADIQDELALNICKDLGSTFVHCDVTKEFDVETAVNTAVSTYGKLDIMLNNAGISGAPKYKISNTQLSDFKRVVDVNLVGVFLGTKHAARVMIPNRSGSIISTASAATAAAAGTPYPYICSKHGVVGLTRNAAVEMGGHGIRVNCVSPYYVATPMTRDDDWIQGCFSNLKGAVLTAEDVAEAALYLASDESKYVSGHNLLVDGGVSIMNQGCNMFDLMDS

>AaBDH

MNGVYPHRLLEGKVAIITGGASGFGESTVRLFAKHGAKVVIADIQDQLGLSLCNDLVNKIGDNVIYLHCDVTKESENIENTVNTAVSKFGKLDIMFNNAGIPGNLDFTILNSDNENFKRVFDVNVFGSFLGAKHAARVMIPAKRGVILFTSSVASVLAGESPHSYTVSKHAVIGLMKNLCVELGQYGIRVNCISPGSVSTPLVTTAMGVDKEVVDGILCASAVLKGVVPTADDVAEAALYLGSDASRYVTGVNLVVDGGYSTTNPTYSRVIKQTFEDLAKKNEGCNGNGVSHAT

>RoBDH1

MSCNTAVSRRLEGKVAIVTGGASGIGASTVRLFHDHGAKVVIADIQDDLGQTLADRLGRNISYTHCDVTDEDQVRALVDAAVAKHGGVDIMFSNAGIVEGPNSIFDVDKDELERLMGINLVGAFLAAKHAARVMVPAKKGCIIFTASACTEIAGIAGHSYTASKYGIVGLMKSLAVELGSHGIRANCVSPFGVLTGIVPDDEASKLMFEGIMSKVGNLKGKILTAEDVAVTVLYLASEEASYVSGVNLLVDGGYTVVNPTFINVITAGQS

>RoBDH2

MKMKPESNGLHTSKRLEGKVAIITGGASGFGEATAALFVRHGAKVVIADVQDDRGSALCRDLGLPNQISYVHCDVTSDADVSAAVDLAVSKYGGLDIMFNNAGIPGGLDFTIVDADNDNFRRVFEVNVYGAFLGAKHAARAMIPARRGGAILFTASVASAVAGESPHSYAASKHAVVGLMRNLCVELGQHGIRVNAISPCAVATPLLTGTMGVEKAVVEDIICASANLKGVVPTAEDVAEAALYLGSDESKFVSGLNLVVDGGYSTTNQSYSRAGTTGSQTLPSASSREPEMG

>Wv02G1602

MLGKLGKRPLLCKKGDLLVRLLIKDVQISYGLYFWLVSRLEGKVAIVTGGARGIGEATVRLFARHGAKVVAADVEDVAGESLAARLGPSVSFVRCDVRQEADIERLVDRTLARHGRLDVFCNNAGVLGRQTPRSDRSIAALDAEEFDGVMRVNVRGAALGMKHAARAMVPRGAGCIISVASVAGVMGGLGPHAYAASKHAIVGLTANAACELGKHGIRVNCVSPFGVATRMLVDAWREVDDAGEHEDVAPADAPPTAEEMEKTEEMVRGLANLKGVTLTVRDVAEAILYLASDESRYVSGHNLVVDGGVTTSRNLIGL

>Wv03G1235

MAVNSRGVFLGTKHAARAMMAAGVRGSIINNGSVATVVAGVASHAYVASKHAVLGLTRSAAAELGQHGIRVNCVSPFLYGTSLACDFIGMDQKRIEEMIGAIGNLKGAVLRGDDVARAAVFLASDESCYVSGQNIIIDGGFTAVSHAFGLFKN

>Wv05G1424

MERRLEGKVAIITGGASGIGEATVKLFVRHGALVIVADVQDEKGKALCAALGSDDVVSYVHCDVRRESDVKRTVDTAISLHGKLDIMFNNAGIVDPAGSSILGEDDTTAVFERVMGVNVLGALLGTKHAGRAMAAVGRGGSIIITASMVSVLGWVGLPAYTCSKHAVVGLARSAAAELGKHGVRVNCVSPTAVATPLAVSALQASEEEVEALGEGMSTLKGVSVKAEDMAKAALFLASDESRFVSGHNLVVDGATTVTKIF

>Wv05G1425

LEGKVAIITGGASGIGEATVKLFVRHGARVIVADVQDEKGKALCAGLGSNDVVSYVHCDVRCESDVSEEEVEALGERLSALKGVRVKAEDMAKAALFLASDESRFVSGHNLVVDGATTVTKIF

>Wv05G1426

LEGKVAIITGGASGIGEATVKLFVSHGARVIVADVQDEKGKALCAGLGSNDVVSYVHCDVRRESDASEEEVEALGERLSTLKGVRVKAEDMAKAALFLASDESRFVSGHNLVVDGASSVTKIF

>Wv05G1427

LEGKVAVITGGASGIGEATVKLFVRHGARVIVADVQDEKGKALCAALGSNDVVSYVHCDVRRESDASEEEVEVFGEGMSTLKGVRAKAEDMAKAALFLASDESRFVSGHNLVVDGASSVTKIF

>Wv05G1429

LEGKVAIITGGASGIGEATVKLFVRHGALVIVADVQDEKGKALCAALGSDDVVSYVHCDVRRESDASEEEVEALGEGMSTLKGVSVKAEDMAKAALFLASDESRFVSGHNLVVDGAATVTKIF

>Wv05G1431

LEGKVAIITGGASGIGEATVKLFVRHGALVIVADVQDEKGKALCAALGSDDVVSYVHCDVRRESDASEEEVEALGEGMSTLKGVSVKAEDMAKAALFLASDESRFVSGHNLVVDGAATVTKIF

>Wv05G1432

LEGKVAVITGGASGIGEATVKLFVRHGARVIVADVQEEKGKALCAGLGSEDVVSYVHCDVRRESDVSEEEVEAMGEVISTLKGVRVKAEDMAKAALFLASDESRFVSGHNLVVDGATTVTKIF

>Wv05G1433

LEGKVAVITGGASGIGEAAVKLFVRHGARVIVADIQDDKGKALCAGLGSDDVVSYVHCDVRLESDASEEEVEAVGEMMSTLKGVRAKAEDIAEAALFLASDESRFVSGHNLVVDGASTVTKVF

>Wv05G1434

LEGKVAVITGGASGIGEATVKLFVRHGARVVVADVQDEKGKALCAGLGSDDAVSYVHCDVRRESDASEEEVEATAEVISTLKGVRAKAKDMAEAALFLASDESRFVSGHNLVVDGAASVTKIF

>Wv05G1435

MGVNVLGAMLGTKHAGRAMVAAGRGGSIIITASVLSVVGWLGLPVYVCSKHAVVGLARSAAAELGKHGVRVNCVSPSVVATPLASSHLQASEEEVEATAEVISTLKGVRAKTKDMAEAALFLASDESRFVSGHNLVVDGASSVTKIF

>Wv05G1436

LEGKVAVITGGASGIGEATVKLFVRHGARVVVADVQDEKGKALCAGLGSDDVVSYVHCDVRRESDASEEEVEATAEVISTLKGVRAKTKDMAEAALFLASDESRFVSGHNLVVDGASSVTKIF

>Wv05G1437

LEGKVAVITGGASGIGEATVKLFVRHGARVVVADVQDEKGKALCAGLGSDDVVSYVHCDVRRESDASEEEVEATAEVISTLKGVRAKTKDMAEAALFLASDESRFVSGHNLVVDGASSVTKIF

>Wv05G1438

MERRLEGKVAIITGGASGIGEATVKLFVRHGALVIVADVQDEKGKALCAALGSDDVVSYVHCDVRRESDVKRTVDTAISLHGKLDIMFNNAGIVDPAGSSILGEDDTTAVFERVMGVNVLGALLGTKHAGRAMAAVGRGGSIIITASMVSVLGWVGLPAYTCSKHAVVGLARSAAAELGKHGVRVNCVSPTAVATPLAVSALQASEEEVEALGEGMSTLKGVSVKAEDMAKAALFLASDESRFVSGHNLVVDGATTVTKIF

>Wv05G1439

PIRTLLSHIKAYKRRDPSSLTKIYLEINKIFSMERRLEGKVAVITGGASGIGEATVKLFVRNGARVVVADVQDEKGKALCAGLGSDDVVSYVHCDVRRESDVKRAVDTAISLYGKLDIMFNNAGILGPTGGSILGEDDTTAVFERVMGVNVLGAMLGTKHAGRAMAAAGRGGARREHHNHGEHGVGAGRAGPSSLHVLEARGGGAGAERGGGAGQAQGACELCVADGGGDAYGGVGPAGERGGGGGIWRGDVDSEGREGEGGGHGKGGAVLGQRRVEVRERPQPRGRRSRQRHQNILNPYLLL

>Wv12G0551

MFRILFRRSATVGREQYCSSPKSVKFYSSAAPPQGGGGLQGKVALVTGGASGLGKATASEFLREGAAAVVLADADARLGQKVAQELGPQADFVECDVTDEPQVAAAVDFAVVRHGRLHVMYNSAGISGPLASPDVSALDLAAFDAVMAANVRGTLAGVKHAARAMGPAGSGSIICVSSVSGIMGGLGTHPYAISKFAVAGIVRSMAGELSRRGVRLNCISPFAIPTPMVLGQFAQIYGGAGTEKLLAIVEGLGELAGAKCEEIDVAKAAVYLASDESKFVSGNNLVVDGGFTSYKQFNMPIPDRI

>Wv13G0704

YRLAGKVAIITGAASGVGKATAAEFIHHGAQAVLADIQHELGKSVAAELGPRATFVPCDVTQEPQVAAVVDLAVAKHGRLDIMYNNAGISGPITFAVTDVDLTEFDRVMAVNVRSVVAGIKHAARVMVPRRAGSILCTASITGFVGGLAPLTYSLSKAAVAAAVRLSASELSKHGIRVNCISPTGLPTPFGLKAIREIFPDLEEQRAVEMIELSTAELAGTKCEVEDVAKAATFLVSDEAKYISGHNLVVDGGFTTFKRLNVSP

>Wv13G0705

RRNHARNGFEVLFNCRLKRGLAIRTGIRTQQQQFSTHPTPPGLAGKVAIITGAASGVGKATAAEFIHRGAQVVLADIQHELGKSVAAELGPCATFVPCDITQESQVAAVVDLAVAKHGRLDIMYNNAGICSPTTYPITDVDLTEFDRVMAVNARSVVAGIKHAARVMVPRRAGSILCTASIAGLVGGLAPLTYSLSKAAVAAAVRLSASDLSKHGIRVNSISPTGMPTPLGLKAIRGIFPDLEEQRAVEVIELCAAELAGTKCEVEDIAKAATFLVSDEAKYISGHNLVVDGGFTTFQRLNVSP

>Wv13G0706

ILHSPCSHLTERNRRRGRVARNHARNGFEVLFNCRLKRGLTIRTGIRTKQQQFSTHPTPGRLDGKVAIITGAASGVGKATAAEFIHRGAQVVLADIQHELGKSVAAELGPCATFVPCDITQESQVAAVVDLAVAKHGRLDIMYNNAGICSPTTYPITDVDLTEFDRVMAVNARSVVAGIKHAARVMVPRRAGSILCTASIAGLVGGLAPLTYSLSKAAVAAAVRLSASDLSKHGIRVNSISPTGMPTPLGLKAIRGIFPDLEEQRAVEVIELCAAELAGTKCEVEDIAKAATFLVSDEAKYISGHNLVVDGGFTTFQRLNVSP

>Wv13G0707

MLGMALRLKRGLAIKTGIRTKQQQFSTHPTPERLAGKVAIITGAASGIGKATAAEFIHRGAQVVLADIQHELGKSVAAELGPCATFVPCDVTQESQVAAVVDLAVAKHGRLDIMYNNAGIWGPTPYAVTDVDLTEFDRVMAVNVRSVVVGIKHAARVMVPRRAGNILCTASIAGLLGGLGPLTYSLSKAAVAAAVRLSASDLNKHGIRVNSISPTGMPTPLGLKAIRGIFLDLEEQRAVEMLELCTSELAGTKCEVEDIAKAATFLVSDEAKYISGHNLVVDGGFTTFQRLNVSP

>Wv13G0710

NIYHSQRQNKIFLYYSSSPTPQIGFKRRPSAGLVLISQRNRRRGRVERNHARNGFEVLFNCRLKRGLAIRTGIRTQQQQFSTHPTPGRLAGKVAIITGAASGVGKATAAEFIHRGAQVVLADIQHELGKSVAAELGPCATFVPCDVTQESQVAAVVDLAVAKHGRLDIMYNNAGIWGPTPYPVTDVDLTEFDRVMAVNVRSVVAGIKHAARVMVPRGAGNILCTSSIAGLLGGLGPLTYSLSKAAVAAAVRLSASDLSKHGIRVNSISPTGMPTPLGLKAIRGIFPDLDEQRAVEMLELCTAELAGTKCEVEDIAKAAAFLVSDEAKYISGHNLVVDGGFTTFQRLNVSP

>Wv16G0089

MEIKRLEGKVAIITGGASGIGEAAVKLFVLHGARVIVADVQDEKGESLCASLGPQGAASYVHCDVRLEADVKRVVDTAISLHGKLDIMFNNAGISDPASSSIVGEGDLTAAFERVMGVNVLGALLGTKHAARAMMASSPPGRGGSIITTTSVASVIGGVAPPVYTCSKHALVGLTRTVAAELGKHGVRVNCVSPALVATPQAVSHSKASKEELEAMGEAISTLKGVKLKAEDIAEAALFLASDESRFVSGHNLVVDGACSVTKLF

>Wv17G0646

MKHAARIMIPKRKGAIVFMGSVSSVIAGAGPHGYTGAKHAVVGLTKSGAAELGKHGIRVNCVSPYAVPARLSMPHLPKSEMQEDALRGFLTFVRSNANLKGVDLMSKDVAEAVLYLASDESKYVSGLNLVVDGGFSIANHTLKVFK

>Wv19G0788

MKRAVRIMIPKRKGSIVFMGSVSSMIAGAGPHGYTGAKHAVVGLTKSGAAELGKHGIRVNCVSPYAVPARLSMPHLPK

>Wv23G0378

MAEARCTLIIIATTLLSSMASSFVLSSLAKRLEGKVALITGGASGVGECTAKLFARLGARVVVADIQDDKGRALCDSLGPDTASYVHCDVTKEPEVASAVDAAVARHGKLDVMFSNAGVSEPMQMFHDCEVADFQRLMSVNVMGAFLATKHAARVMTPARRGSIVITGSTASTIAAGMVPHAYTCSKHAVVGLMRSAAAELGRHGVRVNCVSPHGVATPM

>Wv23G0379

MAETRCTLIIIATTLLSSMASSFVLSSLAKRLEGKVALITGGATGVGECTAKLFARLGARVVVADIQDDKGRALCDSLGPDTASYVHCDVTKEPEVASAVDAAVARHGKLDVMFSNAGVVEPMQMSFHDCEVADFQRLMSVNVMGAFLATKHAARVMTPARRGSIVITGSTASTIAAGMVPHAYTCSKHAVVGLMRSAAAELGRHGVRVNCVSPYGVATPM

>Wv23G0381

MSFHDCEVADFQRLMSVNVMGAFLATKHAARVMTPARRGSIVITGSTASTIAVEMIPHAYTCSKHAVVGLMRSAAAELGRHGVRVNCVSPHGVATPMTVAAFDLDKEGVEALFERSANLKGVRLEAQDVAEAVAYLAGDESRYVSGVNLLVDGGFTIAKELA

>Wv23G0382

MAETRCTLIIIATTLLSSMASSFVLSSVAKRLEGKVALITGGASGFGECTAKLFARLGARVVVADIQDDKGRALCDSLGPDTASYVHCDVTKEPEVASAVDAAVARHGKLDVMFSNAGAVEPITTKKTEIWDEFSDEF
