# Supplementary figures and images for: Multiomics comparison among populations of three plant sources of Amomi Fructus
Source: Hortic Res. 2023 Aug 1;10(8):uhad128. doi: 10.1093/hr/uhad128 (PMC10407604; doi:10.1093/hr/uhad128)

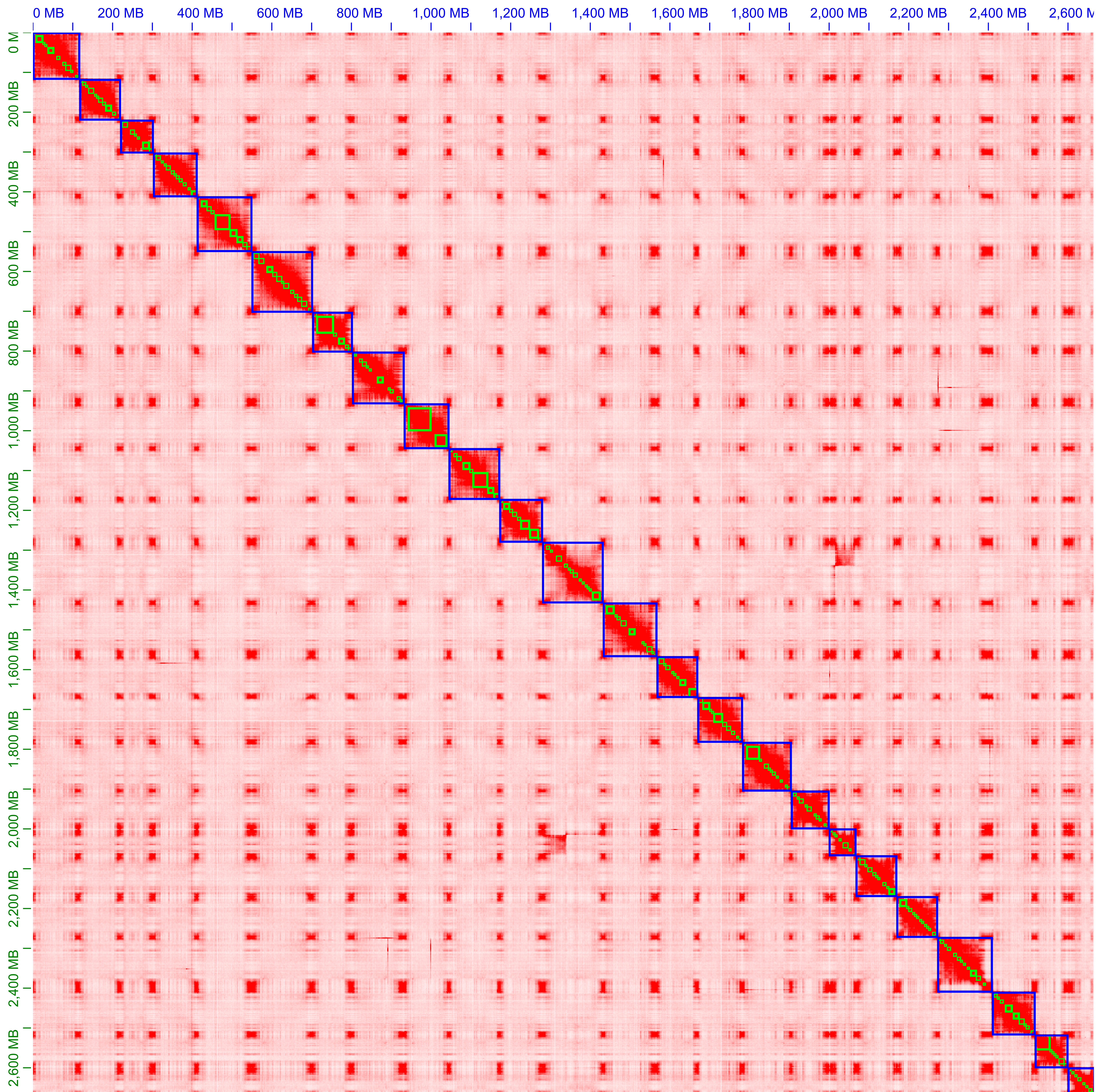

Supplement: Web_Material_uhad128 [file web_material_uhad128.zip › FigS1.pdf]

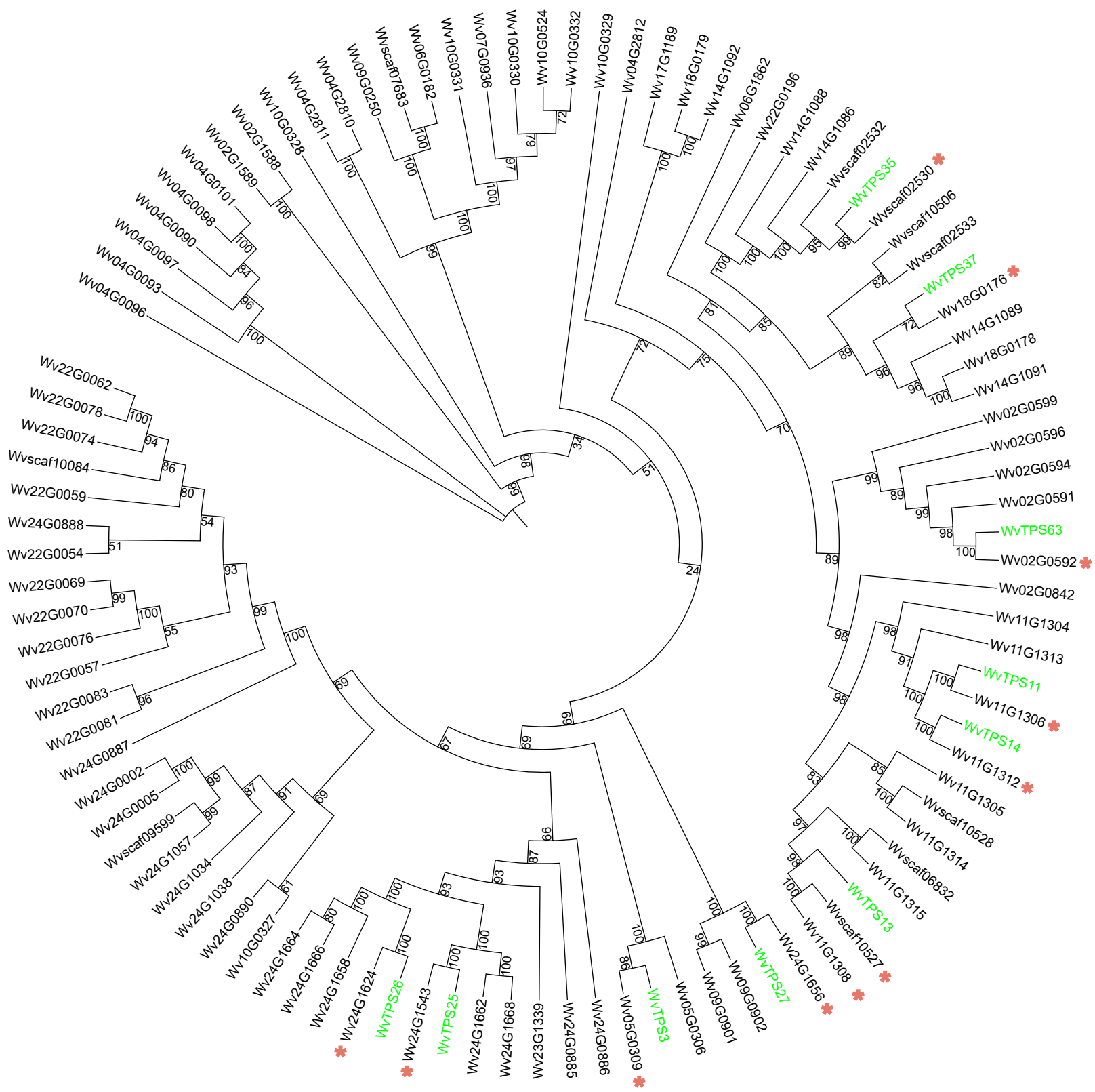

Supplement: Web_Material_uhad128 [file web_material_uhad128.zip › FigS2.pdf]

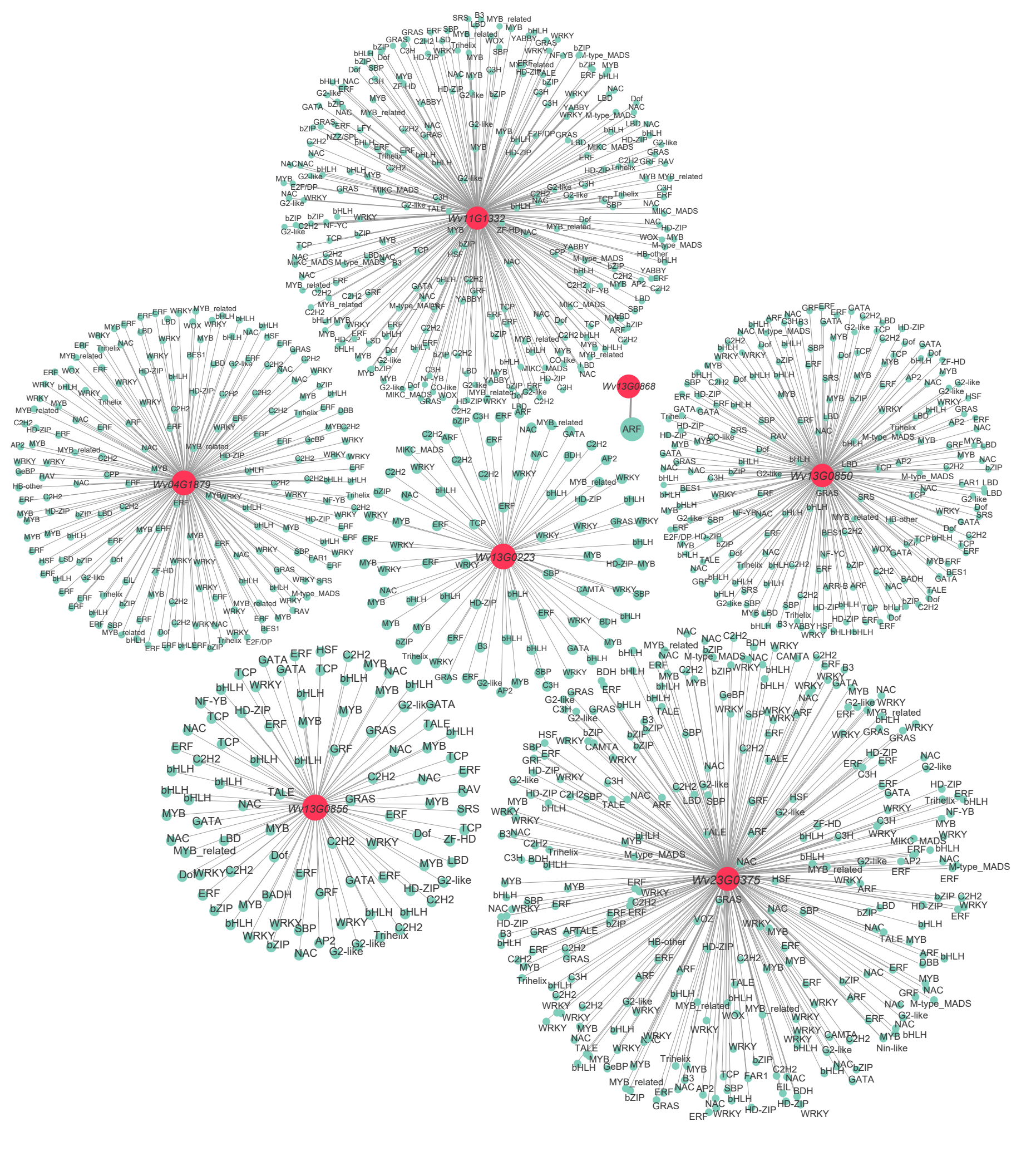

Supplement: Web_Material_uhad128 [file web_material_uhad128.zip › FigS3.pdf]

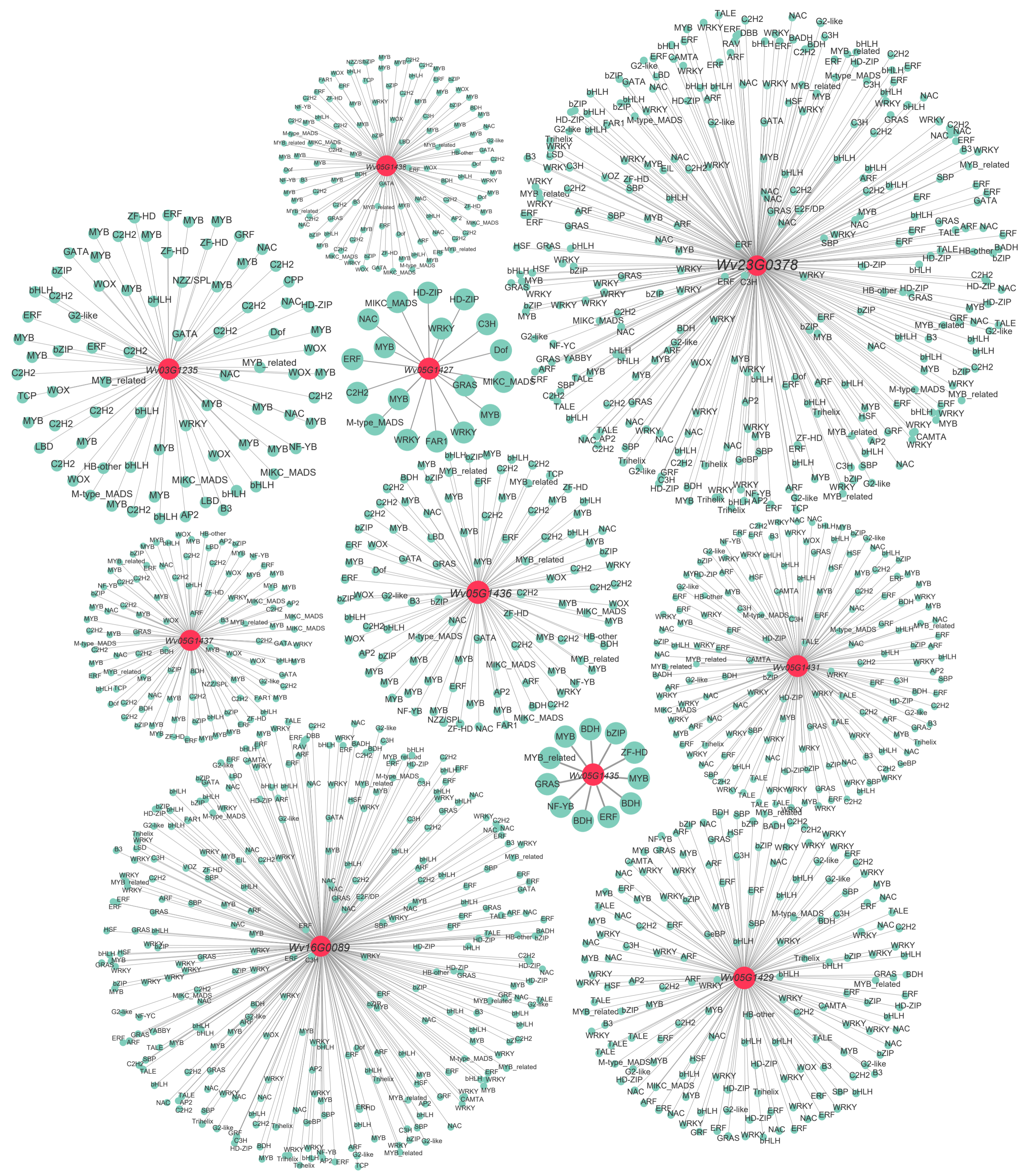

Supplement: Web_Material_uhad128 [file web_material_uhad128.zip › FigS4.pdf]

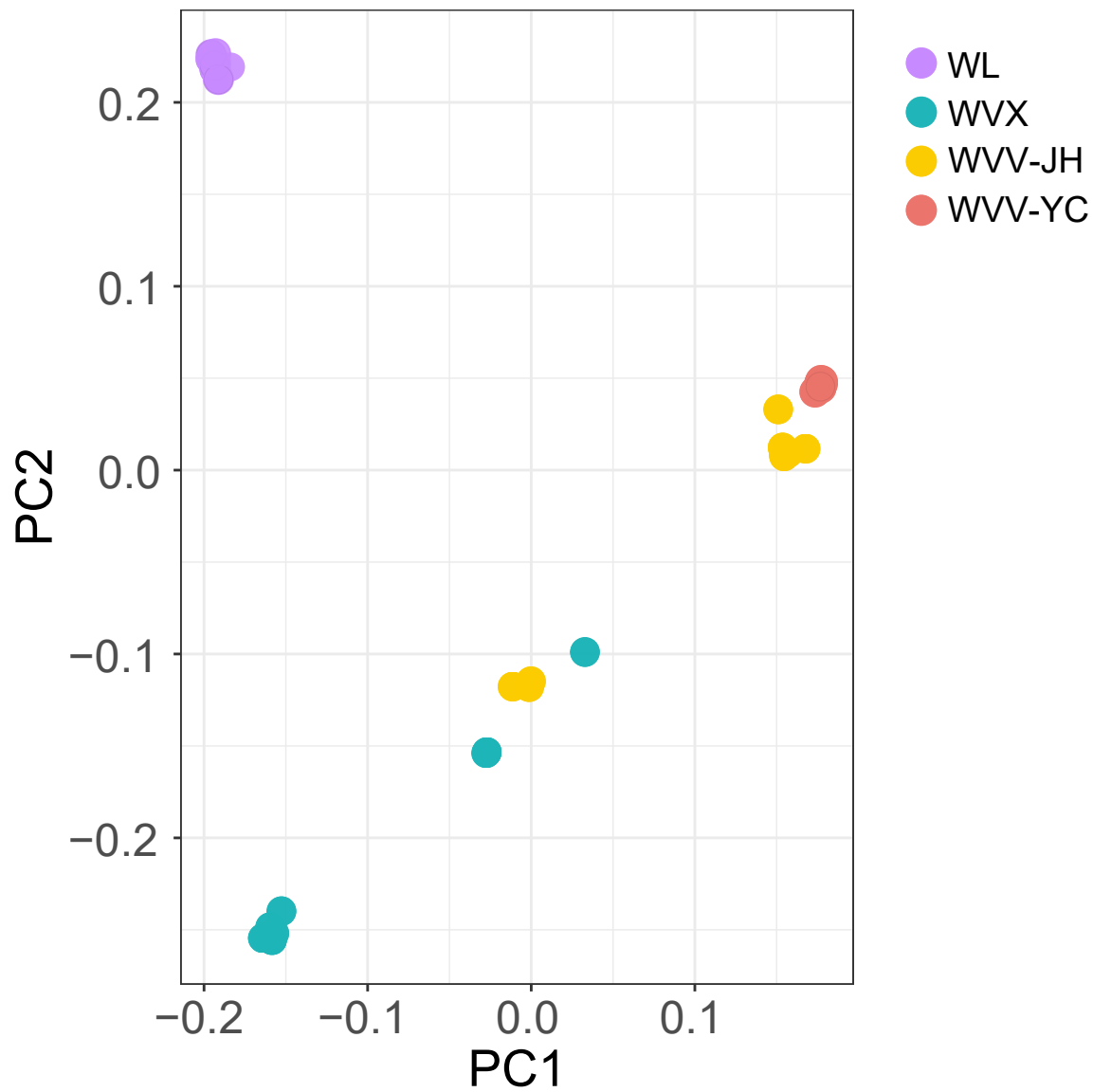

Supplement: Web_Material_uhad128 [file web_material_uhad128.zip › FigS5.pdf]

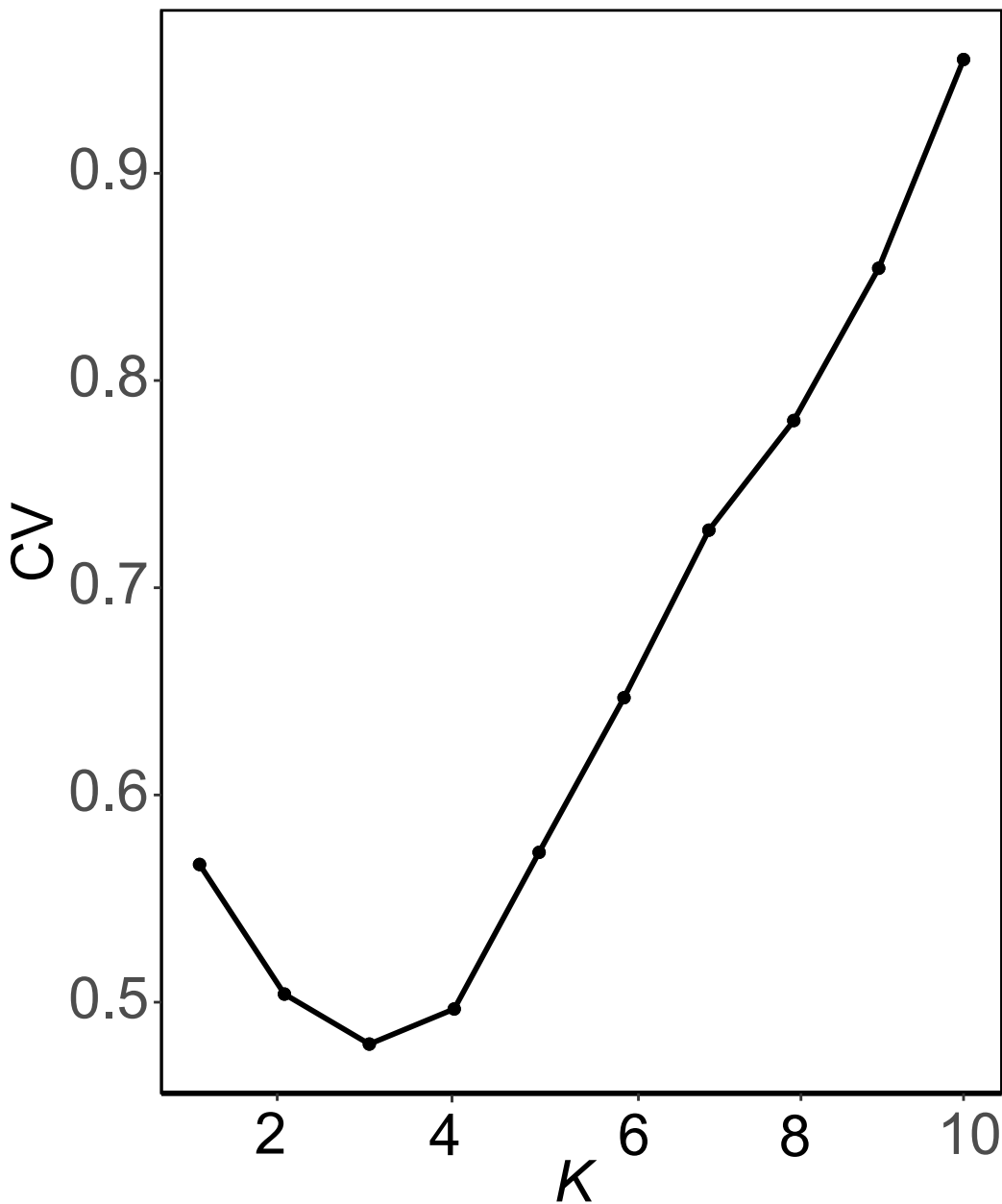

Supplement: Web_Material_uhad128 [file web_material_uhad128.zip › FigS6.pdf]

WVV vs. WVX

WVV-YC vs. WVV-JH

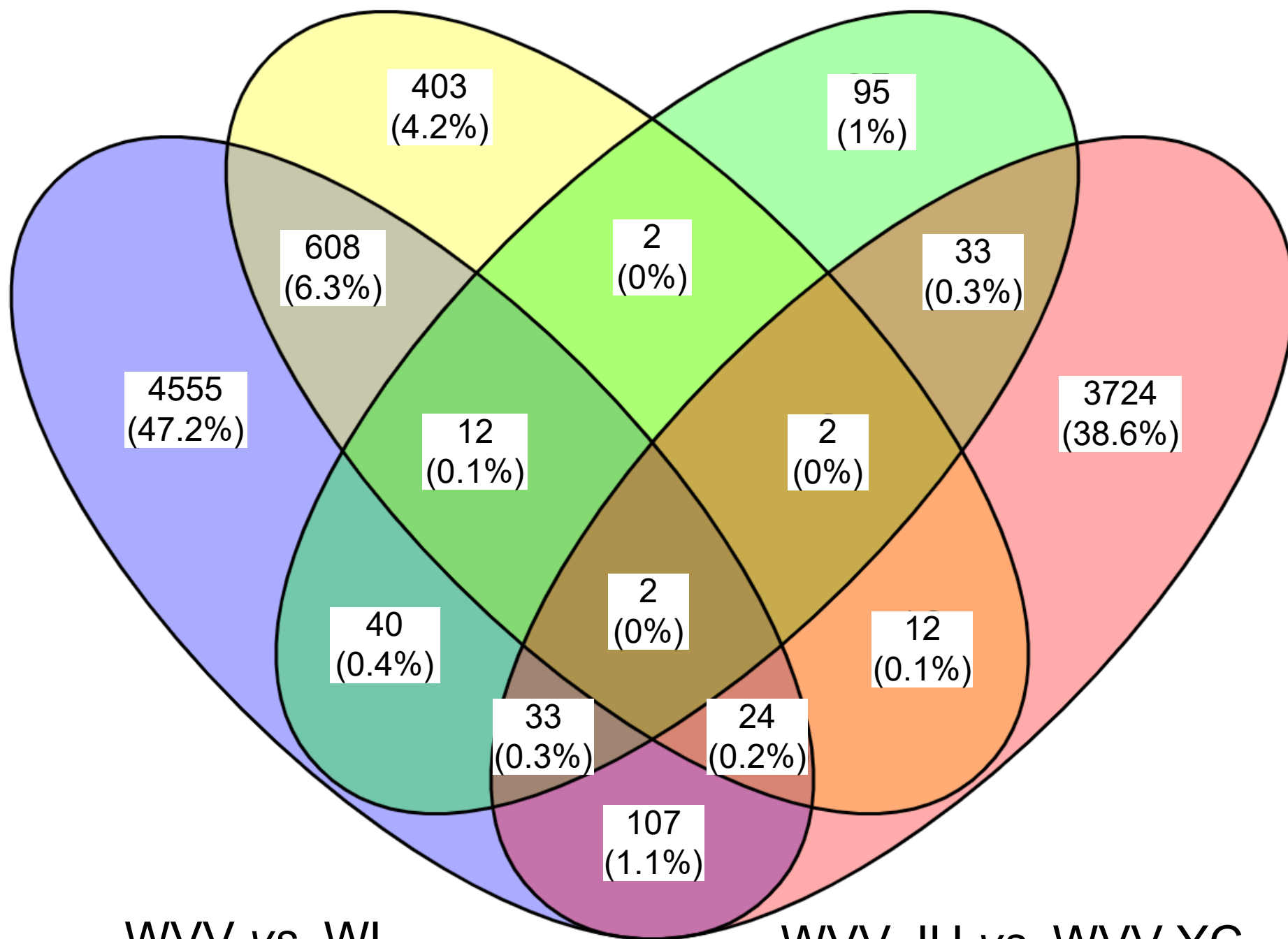

WVV vs. WL

WVV-JH vs. WVV-YC

Supplement: Web_Material_uhad128 [file web_material_uhad128.zip › FigS7.pdf]

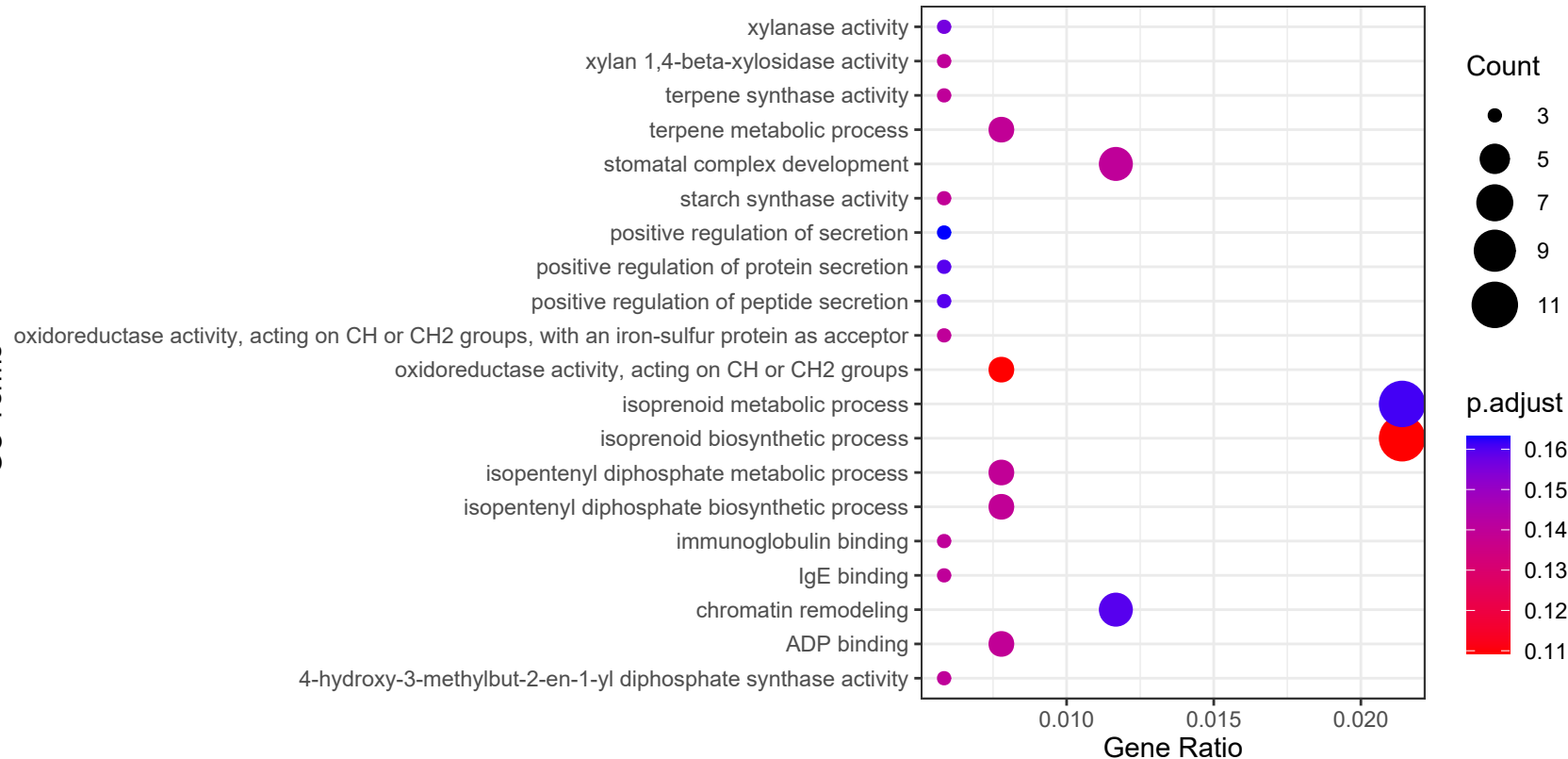

Supplement: Web_Material_uhad128 [file web_material_uhad128.zip › FigS8.pdf]

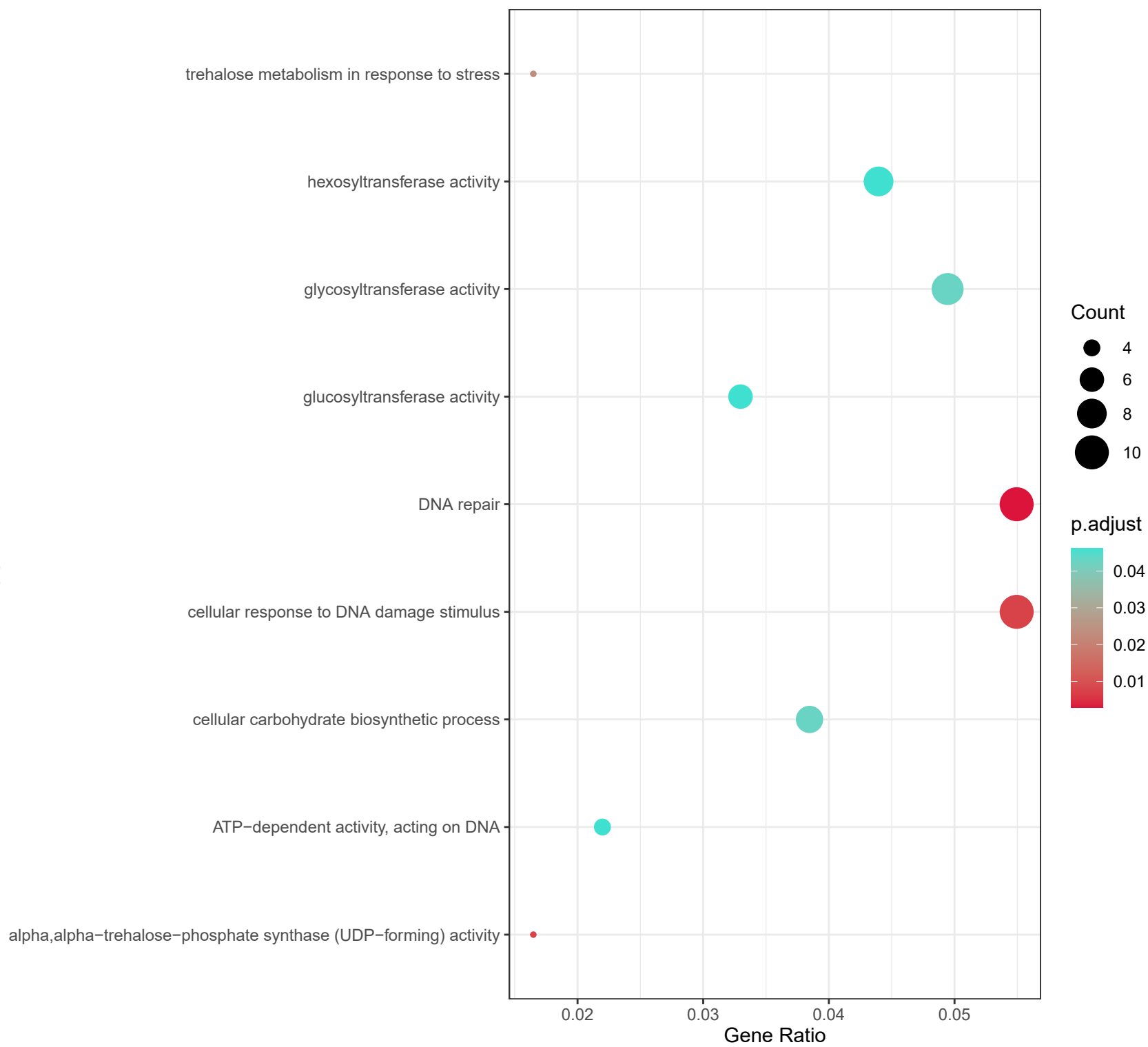

Supplement: Web_Material_uhad128 [file web_material_uhad128.zip › FigS9.pdf]
